# Spatial compartmentalization at the nuclear periphery characterized by genome-wide mapping

**DOI:** 10.1186/1471-2164-14-591

**Published:** 2013-08-30

**Authors:** Feinan Wu, Jie Yao

**Affiliations:** 1 Department of Cell Biology, Yale University School of Medicine, 333 Cedar Street, New Haven, CT 06520, USA

**Keywords:** Nuclear periphery, Nuclear lamina, DamID, High-throughput sequencing, Histone modification, Transcription

## Abstract

**Background:**

How gene positioning to the nuclear periphery regulates transcription remains largely unclear. By cell imaging, we have previously observed the differential compartmentalization of transcription factors and histone modifications at the nuclear periphery in mouse C2C12 myoblasts. Here, we aim to identify DNA sequences associated with the nuclear lamina (NL) and examine this compartmentalization at the genome-wide level.

**Results:**

We have integrated high throughput DNA sequencing into the DNA adenine methyltransferase identification (DamID) assay, and have identified ~15, 000 sequencing-based Lamina-Associated Domains (sLADs) in mouse 3T3 fibroblasts and C2C12 myoblasts. These genomic regions range from a few kb to over 1 Mb and cover ~30% of the genome, and are spatially proximal to the NL. Active histone modifications such as H3K4me2/3, H3K9Ac and H3K36me3 are distributed predominantly out of sLADs, consistent with observations from cell imaging that they are localized away from the nuclear periphery. Genomic regions around transcription start sites of expressed sLAD genes display reduced association with the NL; additionally, expressed sLAD genes possess lower levels of active histone modifications than expressed non-sLAD genes.

**Conclusions:**

Our work has shown that genomic regions associated with the NL are characterized by the paucity of active histone modifications in mammalian cells, and has revealed novel connections between subnuclear gene positioning, histone modifications and gene expression.

## Background

The nuclear periphery is a unique subcompartment of the nucleus that consists of the inner nuclear membrane (INM) and its associated proteins, nuclear lamina (NL) and chromatin [1,2]. A number of genes were found to preferentially localize to the nuclear periphery, and re-position upon transcription activation or cellular differentiation [3-5]. Nonetheless, how gene positioning to the nuclear periphery confers regulatory functions remains largely unclear. Tethering reporter genes to the nuclear periphery led to repression of some, but not all, transgenes as well as several proximal native genes, indicating that roles for the nuclear periphery in transcription are not obligatory but rather occur on a gene-by-gene basis [6-10]. Characterizing the features of chromatin at the nuclear periphery will help to better understand the mechanistic links between subnuclear gene positioning and transcription regulation.

Interactions of chromatin with the NL have been studied by microscopic approaches [11], and alterations to chromatin at the nuclear periphery are often associated with aging [12] or with the presence of lamin mutants [13]. Recently, genome-wide mapping has become a powerful approach in identifying chromatin domains associated with the NL. In particular, DNA adenine methyltransferase identification (DamID) assay has successfully identified large lamina-associated domains (LADs) covering about 40% of the genome in human and mouse cells [14,15]. DamID assay using INM proteins such as Emerin yielded binding profiles that were similar to Lamin B1 [14], indicating that the identified genomic regions largely represent those regions that are preferentially positioned to the nuclear periphery. In mouse cells, LADs are 40 Kb to 15 Mb in size and have lower gene densities, lower transcription activities, and lower occupancy of active histone modifications than non-LAD regions of the genome [15]. The boundaries of LADs are enriched with CTCF binding sites, CpG islands, and promoters transcribing away from the LADs [14]. A recent study has dissected a LAD at the immunoglobulin heavy chain locus and identified DNA sequences that can direct transgenes to the NL in mouse fibroblasts. These sequences are enriched with a GAGA motif and associated with transcription corepressor cKrox and histone deacetylase HDAC3 [16]. While LADs are generally considered as repressive chromatin domains, it is not clear how these domains play regulatory roles on gene transcription.

Cellular imaging and genomic analyses have revealed substantial compartmentalization of the nucleus, and have suggested that this compartmentalization functions in gene regulation [17-19]. Gene transcription at the nuclear periphery is also under the influence of the compartmentalization of chromatin domains and regulatory factors [20-22]. For example, our previous work on the mouse myogenic differentiation system has revealed that the non-canonical TFIID subunit, TAF3, but not other transcription components such as RNA polymerase II (Pol II), TAF4 and TBP, is localized away from the nuclear periphery where the key myogenic gene *MyoD* is located [23]. This differential localization is correlated with the differential occupancy of TAF3 versus TFIID at the *MyoD* promoter. Additionally, we noticed that H3K4me3 — the histone modification labeling active promoters — was localized away from the nuclear periphery, and that recognition of H3K4me3 appeared to be required for the subnuclear localization of TAF3 [23]. Another recent study in the myogenic system revealed that transcription repression by Msx1 required recruiting Polycomb to the nuclear periphery and the enrichment of the repressive histone modification H3K27me3 on target genes [24]. Thus, it would be very interesting to further characterize the features of chromatin at the nuclear periphery by examining localizations of additional histone modifications and their genome-wide distributions relative to DNA sequences associated with the NL. The recent development of high throughput DNA sequencing [25] may reveal additional features of lamina-associated regions with improved sensitivity and resolution. We anticipate that bridging cellular imaging and molecular mapping approaches will present a unique opportunity to cross-examine the compartmentalization of histone modifications at both subcellular and genome-wide levels.

In this paper, we have generated the initial maps of genome-NL interactions in mouse 3T3 fibroblasts and C2C12 myoblasts by DamID assay coupled to high throughput DNA sequencing. We have identified sequencing-based LADs (sLADs) that cover ~30% of the genome and represent the genomic regions with significant NL association. In 3T3 fibroblasts, the sLADs largely overlap with the LADs reported previously [15], and range from a few kb to over 1 Mb in size. These newly constructed high-resolution DamID maps allow the examination of NL association within the structure of genes. For example, we have detected substantially lower NL association around the transcription start sites (TSSs) of expressed sLAD genes. Furthermore, by cell imaging and genome analyses, we have confirmed that several active histone modifications, such as H3K4me2, H3K9Ac and H3K36me3, are localized away from the nuclear periphery and are predominantly distributed outside of sLADs. These findings further support the notion that chromatin at the nuclear periphery is characterized by the paucity of active histone modifications, which may have implications on how peripheral gene positioning affects transcription regulation in mammalian cells.

## Results

### Detecting DNA sequences associated with the NL by DamID coupled to high throughput DNA sequencing

The published genome-NL interaction maps were constructed using DamID assay followed by analysis on genome tiling arrays [14,15] (referred to as Dam-chip hereafter). In this study, we developed the Dam-seq method that couples next-generation sequencing (NGS) to DamID assay. First, we tethered mouse Lamin B1 to *E. coli* DNA adenine methyltransferase (Dam) and expressed the fusion protein in mouse cells. In eukaryotic cells that lack endogenous Dam proteins, the tethered Dam is capable of methylating adenines in GATC sequences that are in close spatial proximity [26]. This fusion protein was successfully used to identify genomic regions associated with the NL in mouse cells [15], and we have confirmed its localization at the nuclear periphery by immunofluorescence staining (not shown). Next, we infected 3T3 mouse embryonic fibroblast (MEF) cells and C2C12 myoblast cells with the lentivirus expressing Dam-Lamin B1 at a very low level [27], purified the genomic DNA from infected cells, specifically amplified the methylated DNA fragments and sequenced them directly. The same protocol was applied to cells expressing free Dam proteins and the sequencing data served as a control to correct for local DNA accessibilities [27] and sequencing biases. We prepared two replicates for each fusion protein (or free Dam) in each cell type and sequenced the DNA libraries separately. Only sequencing reads that were uniquely mapped to chromosome 1–19 and X were retained, and potential PCR duplicates were removed. All pairs of replicates have highly correlated read densities along the genome (Pearson correlation coefficients >0.90, p-value <2e-16) and thus were combined for the follow-up analyses. In total, we obtained 43.3 million and 49.0 million reads from 3T3 MEF cells expressing Dam-Lamin B1 (MEF LmnB1) and free Dam (MEF Dam), respectively, and obtained 66.3 million and 41.9 million reads from C2C12 myoblast cells expressing Dam-Lamin B1 (myoblast LmnB1) and free Dam (myoblast Dam), respectively.

### Construction of genome-NL interaction maps

**Figure 1:**
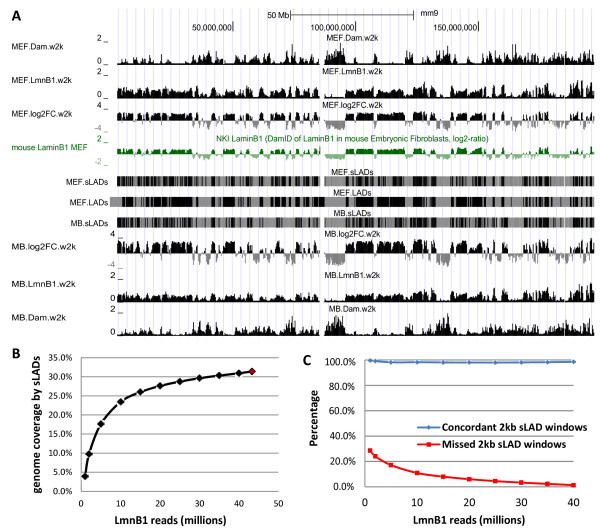
**sLADs/LADs in MEFs and myoblasts (MB). (A)** Chromosome 1 is presented in UCSC genome browser (see Additional file 2: Figure S1 for the entire map; [30]). Tracks “Dam.w2k” and “LmnB1.w2k” plot read densities (RPKM) in non-overlapping, contiguous 2 kb windows along the chromosome. Similarly, the track “log2FC.w2k” plots log2 RPKM ratios of LmnB1 over Dam at a 2 kb resolution. The track “mouse LaminB1 MEF” is taken from the published data [15] which plots the log2 fluorescent signal ratio of LmnB1 over Dam for each microarray probe. Tracks “sLADs” and “LADs” depict the LADs/sLADs (black regions), non-LADs/non-sLADs (grey regions) and undetermined regions (blank regions). **(B)** Estimate of sequencing depths. Random samples of various sizes are generated from 43.3 million of MEF LmnB1 reads, each with three replicates. The plotted genome coverage by sLADs at a sequencing depth is averaged from the three replicates. The red data point represents the result based on the full dataset of 43.3 million reads. **(C)** Estimate of concordance of sLADs identified at different sequencing depths. The sampled sequencing depths are the same set as **(B)**. Concordant 2 kb sLAD windows are 2 kb windows that are identified as sLADs at both the given lower sequencing depth and the highest sequencing depth (the full dataset), and the corresponding percentage is to divide the number of concordant 2 kb sLAD windows by the total number of 2 kb sLAD windows identified at the given sequencing depth. Similarly, missed 2 kb sLAD windows are those identified as non-sLADs at the given lower sequencing depth but as sLADs at the highest sequencing depth, and the percentage is to divide the number of missed 2 kb sLAD windows by the total number of 2 kb non-sLAD windows at the given sequencing depth.

The LADs identified by Dam-chip are large genomic regions ranging from tens of kilo bases to over 10 million bases [14,15]. To identify such broad domains based on the NGS reads, we employed the bioinformatic software SICER [28], which has been successfully used to map histone modifications and their binding proteins [28,29]. Briefly, SICER divides a genome into non-overlapping, contiguous windows, identifies individual windows or larger domains (a set of contiguous windows) in which the experimental sample has significantly higher read counts than the random background (modeled by a Poisson distribution), and calculates the statistical significance of the enrichment by taking into account the control sample. As a result, we have identified 14,737 sLADs (corresponding to 31.4% of the mouse genome) in 3T3 MEFs and 14,828 (32.6% in genome coverage) in C2C12 myoblasts, with the size ranging from 2kb to over 1Mb (Table 1, Additional file 1: Table S1-S2, Figure 1A and Additional file 2: Figure S1). In addition, 9.0% and 8.7% of the genome in MEFs and myoblasts respectively had neither LmnB1 nor Dam reads, and were termed “undetermined regions” that usually corresponded to genome assembly gaps or highly repetitive DNA segments.

**Table 1:** Summary and statistics of LADs/sLADs in MEFs and myoblasts

**Genomic regions**	**LADs/sLADs**						**Non-LADs /non-sLADs genome coverage (%)**	**Undetermined regions genome coverage (%)**
	**LADs/ sLADs genome coverage (%)**	**Number**	**Min. size (Kbp)**	**Max. size (Kbp)**	**Median size (Kbp)**	**Mean size (Kbp)**		
**MEF LADs**[15]	44.4%	1189	59.6	15900	534	991	55.6%	NA
**MEF sLADs**	31.4%	14737	2	1448	34	56.2	59.6%	9.0%
**Myoblast sLADs**	32.6%	14828	2	1090	38	58.1	58.7%	8.7%

We have validated the Dam-seq method by the following analyses using the data from 3T3 MEFs. First, to estimate the depth of sequencing, we identified sLADs from randomly sampled sequencing reads and plotted the sLAD genome coverage versus the number of sampled reads (Figure 1B). The increment of sLAD genome coverage was less than 1% of the genome per five million reads when the input LmnB1 reads were over 25 million, and the genome coverage was close to saturation with ~40 million LmnB1 reads (Figure 1B). This simulation result suggests that the vast majority of the sLADs have been identified with the current sequencing depth (43.3 million 3T3 LmnB1 reads and 66.3 million C2C12 LmnB1 reads). To examine whether the sLADs identified at lower sequencing depths were concordant with those identified at the highest sequencing depth (the full dataset), we plotted percentages of concordant and missed 2 kb sLAD windows at each sampled sequencing depth (Figure 1C). Over 98% of the 2 kb sLAD windows identified at each lower sequencing depth stay as sLADs based on the full dataset, while the percentage of missed 2 kb sLAD windows decreases quickly as the sequencing depth gets higher (Figure 1C). Therefore, increasing sequencing depth retains the vast majority of the sLADs and helps to identify sLADs that are missed at a lower sequencing depth.

Second, similar to the Dam-chip method that measures NL association by log2 fluorescent intensity ratios of LmnB1 over Dam (log2 chip ratios) [14], we computed log2 read density (RPKM, reads per kilo base per million mapped reads) ratios of LmnB1 over Dam (log2 seq ratios) for all the non-overlapping, contiguous 2 kb windows along the genome. The chromosome-wide profiles of log2 seq ratios and log2 chip ratios are fairly similar (Figure 1A, Spearman correlation coefficient 0.74 and p-value <2e-16). The log2 seq ratios range from −7.3 to 7.7 (−4.0 to 3.8 for 5 to 95 percentiles) while the log2 chip ratios range from −5.4 to 4.9 (−1.6 to 1.9 for 5 to 95 percentiles), suggesting that the sequencing method reports a higher dynamic range of NL association.

**Figure 2:**
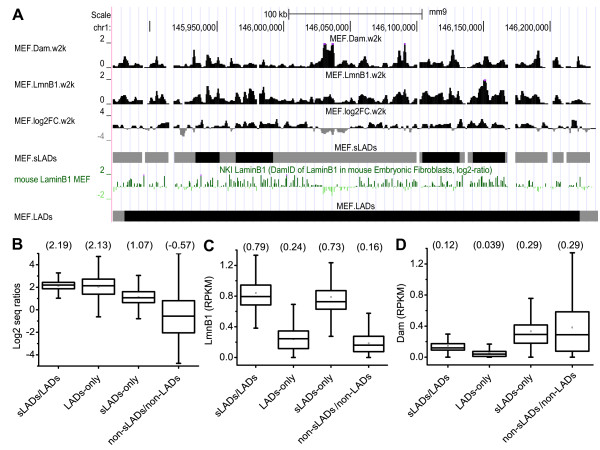
**Comparisons of sLADs and LADs in MEFs. (A)** A 400 kb segment in chromosome 1 shows that a LAD region is comprised of an array of sLADs and non-sLADs. Genome browser tracks follow the same scheme displayed in Figure 1A. **(B**-**D)** Bar plots of **(B)** log2 seq ratios, **(C)** LmnB1 read densities (RPKM) and (**D**) Dam read densities (RPKM) among genomic regions that belong to both LADs and sLADs (common sLADs/LADs), LADs but not sLADs (LAD-only regions), sLADs but not LADs (sLAD-only regions) and neither LADs nor sLADs (common non-sLADs/non-LADs). The upper and lower box boundaries indicate the 25th and 75th percentiles of the data, respectively. The upper and lower bars indicate the first and 99th percentiles, respectively. The median values are shown in parentheses.

Third, we compared the genomic distributions of sLADs and the published LADs in MEFs [15]. The vast majority of LADs are in common with sLADs except that 94 LADs (56.7 Mb, corresponding to 2.1% of the genome and 4.8% of the LAD regions) do not overlap with any sLADs. In many cases, one LAD comprises an array of sLADs and non-sLADs (an example shown in Figure 2A), which is consistent with the smaller sizes of sLADs (Table 1). Our sequencing method also identified sLAD specific regions that cover 108 Mb, 4.1% of the genome and 13.0% of the sLADs. In order to evaluate the differences between sLADs and LADs, we have compared the log2 seq ratios among the following four sets of genomic regions: the common regions between sLADs and LADs (common sLADs/LADs), the regions specific to LADs (LAD-only regions), the regions specific to sLADs (sLAD-only regions) and the regions that are neither LADs nor sLADs (common non-sLADs/non-LADs) (Figure 2B). Pairwise comparisons using Wilcoxon-Mann–Whitney tests have shown that the median log2 seq ratios decrease in the above order (p-values <0.01 after Bonferroni correction for multiple hypothesis tests). Further examination of the LmnB1 and Dam read densities in these datasets has revealed that the median LmnB1 read density in LAD-only regions is only slightly higher than that in common non-sLADs/non-LADs and is much lower than those in common sLADs/LADs and sLAD-only regions (Figure 2C). Also, the median Dam read density in LAD-only regions is the lowest among the four datasets (Figure 2D). Therefore, LAD-only regions were excluded by SICER due to low LmnB1 read densities. These regions might have fairly high LmnB1/Dam ratios, but low LmnB1 and Dam read densities might introduce false positives. In contrast, sLAD-only regions were retained by SICER because of high LmnB1 read densities and statistically significant LmnB1/Dam ratios (Figure 2B-C). To summarize, SICER identifies 2 kb windows with high LmnB1 read densities, retains regions with statistical significance by comparing with Dam read densities, and includes gap regions (a maximum of 6 kb in our case) that may not have high LmnB1/Dam ratios or high LmnB1 read densities, in order to account for the potential lack of sequencing depth. The last step partly explains the overlapping data ranges among these four datasets (Figure 2B-D). We note that some LAD-only regions may be identified as sLADs at a higher sequencing depth, but the ability of SICER to recognize the heterogeneity in LmnB1/Dam ratios within LADs is important to compare NL association at a finer scale.

### Validation of the subnuclear localizations of sLADs and sLAD genes

**Figure 3:**
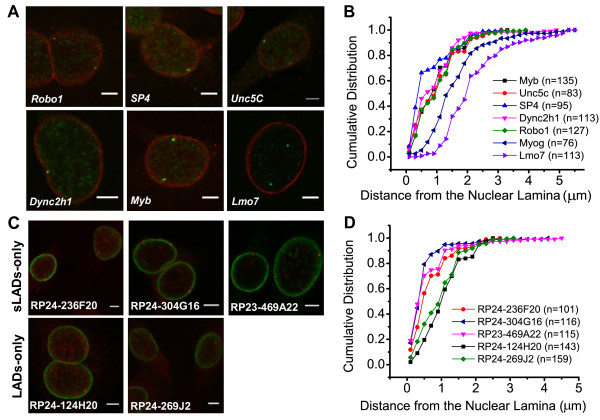
**FISH validation of the nuclear localizations of sLAD and non-sLAD genes. (A)** Representative DNA FISH images of five sLAD genes (*Robo1*, *Sp4*, *Unc5c*, *Dyn2h1* and *Myb*) and a non-sLAD gene (*Lmo7*) in C2C12 myoblasts. Lamin B was labeled red and FISH signals were labeled green. Scale bars are 5 μm. **(B)** Cumulative distributions of the distances of FISH signals in (**A**) relative to the NL. Kolmogorov-Smirnov (K-S) test of FISH signal distances to the NL between any of the sLAD genes and *Lmo7* (a non-sLAD gene): p < 0.001. *MyoG* was reported previously to be localized at the nuclear interior [23] and is also shown here as a reference. **(C)** Representative DNA FISH images of three sLAD-only regions and two LAD-only regions in 3T3 MEF cells. Lamin B was labeled green and FISH signals were labeled red. Scale bars are 5 μm. Genomic locations for these FISH probes are shown in Additional file 2: Figure S2. **(D)** Cumulative distributions of the distances of FISH signals in **(C)** relative to the NL. K-S test of FISH signal distances to the NL between sLAD-only regions and LAD-only regions: p < 0.001 in all cases (i.e., tests between any of the three sLAD-only regions and any of the two LAD-only regions).

By DNA fluorescence in situ hybridization (FISH), we validated the spatial proximities of sLADs relative to the NL. First, we examined the localizations of several gene-containing regions in sLADs and non-sLADs in C2C12 myoblasts. We defined sLAD genes as those with more than 60% sLAD coverage over the gene body, i.e. from TSS to transcription end site (TES) (Additional file 1: Table S3). Indeed, all five sLAD gene loci that we have tested are localized preferentially to the nuclear periphery in C2C12 myoblasts (Figure 3A), among which 40–60% of the loci are located within 0.5 μm to the NL (Figure 3B). Given that the mobility of gene loci is usually constrained to about 0.5 μm in mammalian cells [31], it is likely that these gene loci are contacting the NL at a significant frequency. In sharp contrast, non-sLAD genes are located away from the NL (Figure 3A), and the fraction of gene loci located within 0.5 μm to the NL is below 5% (Figure 3B). Therefore, FISH imaging analyses have confirmed the proximities of these sLAD genes to the NL in C2C12 myoblasts.

Next, we compared the subnuclear localizations of three sLAD-only regions and two LAD-only regions in 3T3 fibroblasts (Figure 3C-D and Additional file 2: Figure S2). All three sLAD-only regions were confirmed to be spatially proximal to the NL (50%–80% of loci located within 0.5 μm to the NL, Figure 3C-D), while the two LAD-only regions were found to be less proximal to the NL (20–30% of loci located within 0.5 μm to the NL, Figure 3C-D). Therefore, at least some of the sLAD-only regions (not identified as LADs by the Dam-chip method) are indeed spatially proximal to the NL and some LAD-only regions (not detected as sLADs by our Dam-seq method) are less proximal to the NL. In summary, all the above FISH analyses have supported the validity of our newly introduced Dam-seq method in identifying genomic regions that are positioned close to the NL.

### NL association across genic regions in myoblasts

**Figure 4:**
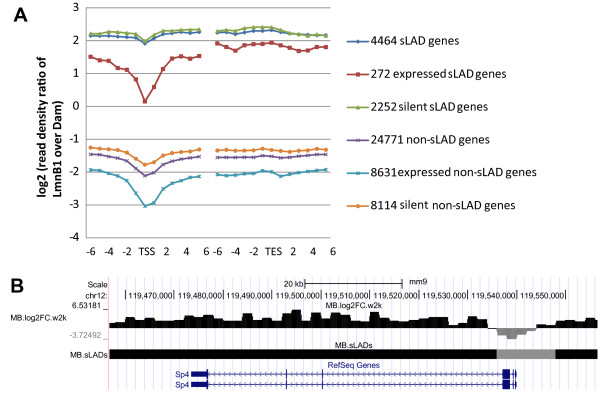
**Nuclear lamina association profiles across genic regions. (A)** The entire set of 29235 genes was assigned to two groups: sLAD genes and non-sLAD genes. Among them, 19269 genes with expression data [32] were further assigned to four subgroups: expressed sLAD genes, silent sLAD genes, expressed non-sLAD genes and silent non-sLAD genes. Log2 seq ratios of genes in the same group were aligned using the 2 kb windows containing TSSs and TESs respectively, and the average log2 seq ratios of the six 2 kb windows upstream, the window containing TSSs/TESs and the six 2 kb windows downstream were plotted. **(B)** A genome browser view of the *Sp4* gene expressed in C2C12 myoblasts and its NL association status.

As described earlier, we have computed genome-wide NL association as log2 seq ratios of LmnB1 over Dam for 2 kb windows along the genome. This dataset has allowed us to characterize the NL association across genic regions in C2C12 myoblasts. The average NL association across sLAD genes are much higher (3 ~ 4 folds in terms of log2 seq ratios) than non-sLAD genes (Figure 4A). Interestingly, NL association is lower in TSS proximal regions compared with the remaining genic regions in both sLAD genes and non-sLAD genes (Figure 4A). At individual gene level, TSS proximal regions of active sLAD genes are often excluded from sLADs and fall into non-sLAD regions (see Figure 4B for an example). Furthermore, 19269 out of 29235 genes with expression data [32] can be grouped into four subcategories: expressed and silent sLAD genes as well as expressed and silent non-sLAD genes. Compared with silent sLAD genes, expressed sLAD genes have more significantly decreased NL association in TSS proximal regions (Figure 4A). We note that TSS proximal regions have a higher read count of free Dam but not LmnB1 than the remaining genic regions and the regions upstream of the genes (Additional file 2: Figure S3), which suggests that TSS proximal regions are not generally refractory to DNA adenine methylation. Instead, they may be more accessible to the free Dam enzyme. We also note that genomic regions around TESs do not appear to have a reduced NL association (Figure 4A). Taken together, our analysis has revealed that TSS proximal regions have decreased NL association, and that the levels of this decrease appear to correlate with gene expression.

### Differential compartmentalization of histone modifications at the nuclear periphery

**Figure 5:**
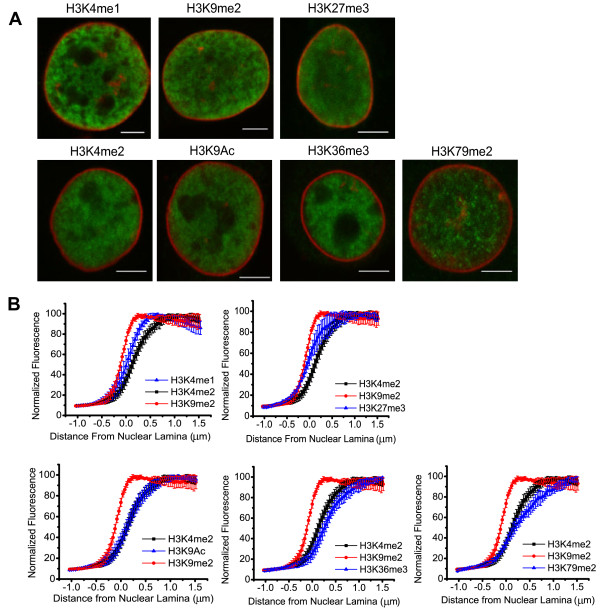
**Localizations of histone modifications relative to the NL. (A)** Representative immunofluorescence staining images of histone modifications (green) and lamin B (red) in C2C12 myoblast nuclei. Scale bars are 5 μm. **(B)** Radial intensity plots integrated over the entire contour of NL from representative cell nucleus images of histone modifications: H3K4me1 (n=6), H3K4me2 (n=6), H3K9Ac (n=7), H3K9me2 (n=4), H3K27me3 (n=6), H3K36me3 (n=8), and H3K79me2 (n=9). Distances from the NL (x-axis labels, in μm) switch from negative to positive values where locations to measure pixel intensities progress from outside the NL to inside the NL, as described previously [23]. H3K4me2 (black) and H3K9me2 (red) intensities were used in each plot to represent histone modifications located away from the NL and located towards the NL, respectively. Error bars represent standard deviations. Two-sample t-test results of the normalized immunostaining fluorescence intensities within 0 to 0.5 μm distance from the NL center are the following: H3K4me2 vs H3K9me2: p < 0.001, H3K4me2 vs H3K27me3: p < 0.01, H3K4me2 vs H3K4me1: p < 0.05, H3K4me2 vs H3K9Ac: p = 0.95, H3K4me2 vs H3K36me3: p < 0.05, H3K4me2 vs H3K79me2: p = 0.08, H3K9me2 vs H3K4me1: p < 0.01, H3K9me2 vs H3K27me3: p < 0.01.

High resolution cell imaging experiments have revealed the localization of a core promoter factor TAF3 and the active histone modification H3K4me3, but not the repressive histone modification H3K9me3, away from the nuclear periphery [23]. Thus, we were interested in probing whether the differential compartmentalization at the NL also applies to additional histone modifications. Using the confocal microscopy imaging method previously described [23], we analyzed the distributions of seven key histone modifications relative to the NL: H3K4me1, H3K4me2, H3K9me2, H3K9Ac, H3K27me3, H3K36me3 and H3K79me2. H3K9me2 is a mark of inactive chromatin and is enriched at the NL [33]; concomitantly, it is located throughout the nucleoplasm including the nuclear periphery, and the half maximum of its immunostaining intensity is positioned about 0.1 μm outside of the center of the NL immunostaining signal (Figure 5). Similarly, H3K27me3, an indicator of the polycomb-repressed chromatin [34], is also located throughout the nucleoplasm including the nuclear periphery, and the half maximum of its immunostaining intensity is approximately at the center of the NL staining signal (Figure 5). In sharp contrast, four active histone modifications – H3K4me2, H3K9Ac, H3K36me3 and H3K79me2 – are all localized substantially away from the NL, with the half maximum of immunostaining intensities to be about 0.15 to 0.25 μm inside of the NL staining signals (Figure 5). The immunostaining pattern of H3K4me1 appears to be intermediate between repressive histone modifications and active histone modifications (Figure 5).

**Figure 6:**
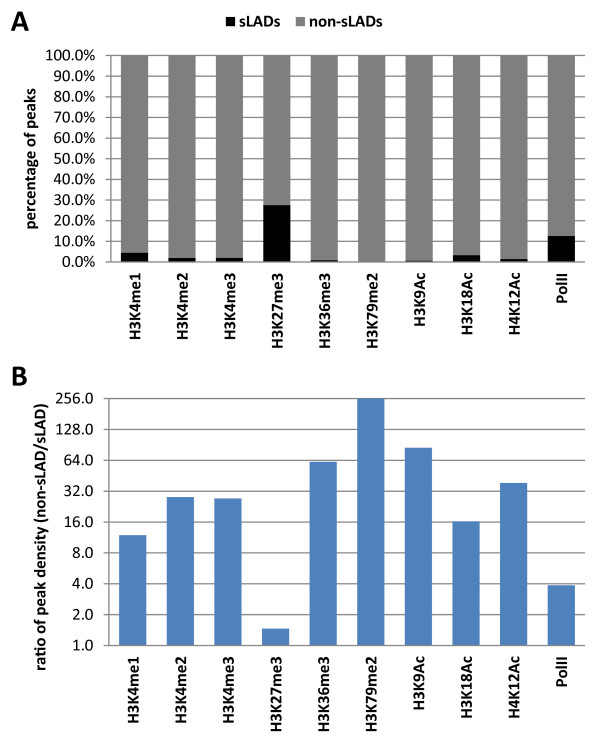
**Distributions of histone modifications and Pol II relative to sLADs in C2C12 myoblasts. (A)** Proportions of ChIP-seq peaks of histone modifications and Pol II within sLADs (black) or within non-sLADs (grey) according to the peak center. ChIP-seq peaks were obtained from [35,37]. **(B)** Ratios of ChIP-seq peak densities in non-sLADs to those in sLADs for each histone modification and Pol II. The peak densities in sLADs and in non-sLADs were computed as the ChIP-seq peak number divided by the genome coverage. Peak density ratios were plotted in the log2 scale at the vertical axis.

Next, we evaluated whether the observed differential compartmentalization of active histone modifications at the nuclear periphery is consistent with their genome-wide distributions relative to sLADs. We performed this analysis using the recently published ChIP-seq data of a number of histone modifications and Pol II in C2C12 myoblasts [35-37]. For all the eight active histone modifications (we examined the subnuclear localizations of five of them as described above), less than 5% of the ChIP-seq peaks are within the sLADs (Figure 6A) and the peak densities in non-sLADs are substantially higher (about 10 to over 200 folds) than those in sLADs (Figure 6B). These findings reveal that active histone modifications are largely distributed outside of sLADs, which is consistent with their localizations away from the nuclear periphery as observed by cellular imaging (Figure 5). In contrast, the repressive histone modification H3K27me3 has nearly 30% of its ChIP-seq peaks distributed within the sLADs (Figure 6A) and its peak density in sLADs is similar to that in non-sLADs (Figure 6B); consistently, H3K27me3 is localized throughout the nucleoplasm including the nuclear periphery as observed by cell imaging (Figure 5). Furthermore, about 12% of Pol II binding sites (detected with the 8WG16 antibody which recognizes the unphosphorylated form of the C-terminal domain of Rpb1) [35] are within sLADs (Figure 6A), and the binding site density in non-sLADs is about four folds of that in sLADs (Figure 6B). Because some of the sLAD genes are actively transcribed, it is expected that a portion of Pol II binding sites are present in sLADs. Consistently, Pol II was localized closer to the NL than active histone modifications as revealed by cellular imaging [23]. Taken together, we have made highly consistent observations in the differential compartmentalization of active and repressive histone modifications relative to the nuclear periphery at the microscopic level and relative to sLADs at the molecular level.

### Distributions of histone modifications among sLAD and non-sLAD genes

**Figure 7:**
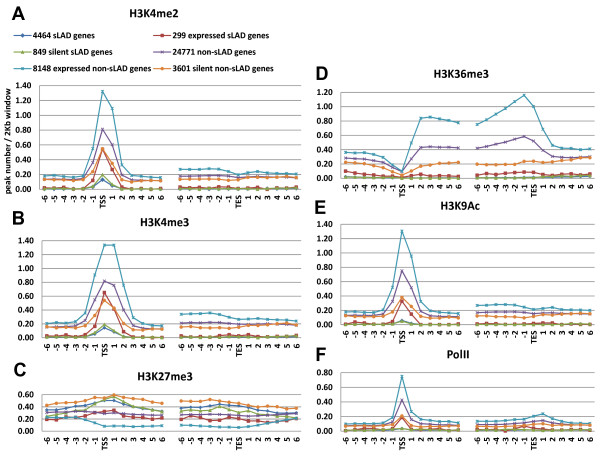
**Distributions of histone modifications and Pol II across genic regions.** ChIP-seq data are the same set as in Figure 6. For the six gene categories described in Figure 4A, peak density profiles of histone modifications were aligned using the 2 kb windows containing TSSs and TESs respectively, and the average peak densities (the peak number per 2kb window) of the six 2 kb windows upstream, the window containing TSSs/TESs, and the six 2 kb windows downstream were plotted: **(A)** H3K4me2, **(B)** H3K4me3, **(C)** H3K27me3, **(D)** H3K36me3, **(E)** H3K9Ac, **(F)** Pol II (8WG16). See Additional file 2: Figure S4 for plots of the other four histone modifications.

Similar to depicting NL association across genic regions for each of the six gene categories (Figure 4A), we have also analyzed the distributions of the above histone modifications and Pol II in the same genomic regions (Figure 7 and Additional file 2: Figure S4). As reported previously [35], active histone modifications as well as Pol II form peaks around TSSs (Figure 7A, B, E, F and Additional file 2: Figure S4) except for H3K36me3, which has a major proportion of its peaks along the gene body (Figure 7D). We have noticed that: 1) both expressed sLAD genes and expressed non-sLADs genes have active histone modifications (such as H3K4me2/3 and H3K9Ac) and Pol II enriched in TSS proximal regions (Figure 7A, B, E, F, and Additional file 2: Figure S4). 2) H3K36me3 levels in the gene body appear to be lower in expressed sLAD genes than expressed non-sLAD genes (Figure 7D). 3) H3K27me3 levels are slightly higher in silent genes than in expressed ones, and the difference is more evident between silent and expressed non-sLAD genes (Figure 7C).

## Discussion

In this study, we have combined the DamID assay and high throughput DNA sequencing to measure genome-NL interactions, and have developed a robust bioinformatic approach that is suitable for identifying genomic regions associated with Lamin B1 (named sLADs). In MEF cells, sLADs largely overlap with the LADs previously identified using the DNA microarray approach [15]. Comparisons of sLADs and gene expression data in MEFs and myoblasts support a negative correlation (Additional file 3), consistent with previous reports [15]. The method demonstrated here is applicable to identifying NL-associated regions in other mammalian cell types or differentiation systems, as well as to identifying genomic regions specifically associated with individual INM proteins and their mutants.

We have noticed that among sLAD genes, NL association is decreased in TSS proximal regions and that the decrease in NL association appears to correlate with gene expression. Highly sensitive, direct measurements of transcription are needed in order to define more precisely the relationship between gene expression and NL association. The reduced NL association in TSS proximal regions can be due to their increased spatial separations from the NL, or their increased chromatin accessibilities, and it is likely that both scenarios may contribute to this observed phenomenon. It is also intriguing to speculate that the reduced NL association in TSS proximal regions may provide a local environment amenable for recruiting transcription machineries and transcription initiation.

We have performed parallel examinations on the nuclear compartmentalization of several key histone modifications in C2C12 myoblasts by cellular imaging and a combined analysis of ChIP-Seq and Dam-Seq data. Histone modifications (such as H3K27me3) that are localized throughout the nucleoplasm including the nuclear periphery are distributed both within and outside of sLADs with similar densities. In contrast, active histone modifications that are largely located away from the nuclear periphery are also predominantly distributed outside of sLADs (Figure 6). These highly consistent results indicate that in C2C12 myoblasts, chromatin at the nuclear periphery is largely characterized by the paucity of active histone modifications. It remains an open question about chromatin features at the nuclear periphery in other cell types such as stem cells and terminally differentiated cells. For instance, it was observed that the repressive H3K27me3 mark is enriched at the nuclear periphery in mouse embryonic stem cells [38]. It would also be interesting to determine whether similar spatial compartmentalization also exists for protein factors associated with these histone modifications (such as TAF3 reported in the previous study [23]). The molecular and imaging methods demonstrated here and in the previous report [23] may be used for future investigations.

## Conclusions

By coupling next generation sequencing to the DamID assay, we have identified sLADs that are genomic regions with significant association to the NL, ranging from a few kb to over 1 Mb and covering about 30% of the mouse genome. Single cell imaging and genomic analyses have provided consistent evidence on the paucity of several active histone modifications at the nuclear periphery both at the microscopic and the molecular levels in C2C12 myoblasts. Comparing sLAD genes and non-sLAD genes has revealed distinct histone modification levels and NL association that correlate with gene expression states. Our work may give clues on how gene positioning to the nuclear periphery (via NL association) affects transcription regulation in mammalian cells.

## Methods

### Cell culture

NIH-3T3 mouse fibroblasts were obtained from ATCC (CRL-1658) and were grown in DMEM + 10% newborn calf serum. C2C12 mouse myoblasts were grown in DMEM + 10% fetal bovine serum. Both cells were grown at a 37°C incubator with 5% CO_2_.

### DamID followed by NGS

DamID was performed using the lentivirus transduction protocol as previously described [27]. Briefly, mouse Lamin B1 cDNA was cloned into the pLGW-dam-V5 vectors kindly provided by the Van Steensel lab. Lentivirus with Dam-V5 and Dam-V5-LmnB1 vectors was generated by the Virapower Lentivirus Expression system (Invitrogen) and was stored at −80°C before use. The lentivirus was diluted with the growth medium and incubated with the cells for two days before changing to the growth media. On day 3 after infection, genomic DNA was isolated using DNeasy blood and tissue kit (Qiagen). The isolated genomic DNA was subject to DpnI digestion, ligation of DamID adaptors and DpnII digestion. Samples without addition of DpnI or adaptors were prepared in parallel as negative controls. Then a 5 μl aliquot of the DpnII digestion was used as template in a 50 μl reaction to amplify methylated genomic fragments.

To prepare for NGS, the above PCR products were purified and digested by DpnII again to remove DamID adaptors, and fragmented by NEBNext® dsDNA Fragmentase (New England Biolabs). Because the methyl PCR products are a smear of 200~2000 bp and the appropriate fragment size for sequencing is ~200 bp, we experimentally determined the appropriate time of fragmentation. For each DNA sample, we performed six reactions, each fragmenting 1μg DNA with 1 μl Fragmentase, incubated them at 37°C for 25 to 50 minutes at an interval of 5 minutes and pooled all for purification. The fragmented DNA was further subject to end repair, addition of “A” bases, ligation of NGS adaptors, gel selection of 300 bp fragments and PCR enrichment according to the protocol provided by Yale Center for Genome Analysis (YCGA, http://ycga.yale.edu/sequencing/Illumina/protocols.aspx) with reagents ordered from NEB. The sequencing was performed by YCGA using Illumina’s HiSeq 2000 sequencing system. The sequencing reads and the alignments (see the next section) have been deposited in the Gene Expression Omnibus (GEO) database (http://www.ncbi.nlm.nih.gov/geo) with accession number GSE41583.

### Mapping of NGS reads

All 75 bp single-end reads passing the quality controls from YCGA were mapped onto the mouse genome assembly mm9 using Bowtie2-2.0.0-beta5 [39] with the following command: bowtie2 –phred3 –× mm9 –U <in.fastq> −S <out.sam>. Only sequencing reads uniquely mapped to chromosome 1–19 and X with mapping quality ≥ 40 were used for further analyses. Potential PCR duplicates were removed using SICER [28]. Read counts in all 10 Kb non-overlapping contiguous windows along the genome were used to estimate the Pearson correlation coefficient of the two biological replicates.

### Identification of sLADs and the related comparative analyses

We used SICER [28] to identify genomic regions enriched with methylated DNA sequences. SICER is a bioinformatic software designed to analyze diffuse ChIP-seq signals. It partitions the genome into non-overlapping, contiguous windows of user-specified size and records the NGS read counts in each window for the sample library. A Poisson distribution (the parameter λ as the average read count per window over the genome) is then used as the background model to score each window and identify “eligible” windows that have read counts significantly higher than the random background. Then SICER combines windows separated by gaps less than a predetermined size in order to compensate for unsaturated sequencing. Next, after normalizing both sample and control libraries to the same size (one million reads), the significance of enrichment is determined for each of the “eligible” windows or domains (a cluster of contiguous windows) relative to the control library. The false discovery rate (FDR) is also calculated to correct for multiple testing.

SICER_v1.1 was downloaded from http://home.gwu.edu/~wpeng/Software.htm, and the script SICER.sh was run with the following parameters: redundancy threshold = 1, effective genome fraction = 0.81, window size = 2000, gap size = 6000, FDR = 0.001, E-value=0.1. We chose the window size of 2 kb based on the fact that the methylation of a Dam protein may spread up to 5 kb from its binding site [26], and chose the gap size of 6000 bp to account for the potential lack of sequencing depth. The effective genome fraction is the uniquely mappable proportion of the genomic sequences of a certain size, and is used to calculate the background parameter λ. The value we chose here corresponds to the 75 bp reads [40]. Based on the SICER-reported normalized read count profiles (the library size was normalized to one million reads) for all non-overlapping, contiguous 2 kb windows along the genome, we termed windows with neither LmnB1 nor Dam reads “undetermined regions” that usually represent genome assembly gaps or highly repetitive DNA segments. It should be noted that these regions contain very few genes or ChIP-seq peaks of histone modifications, thus their exclusion from analysis does not affect the conclusions in the paper. The last window in each chromosome which is smaller than 2 kb was not processed by SICER and therefore was considered as “undetermined regions” too. To calculate the log2 seq ratio for each corresponding 2 kb window (Figure 1A and Additional file 2: Figure S1), we normalized the read density in each window to reads per kilobase per million uniquely mapped reads (RPKM), set a small pseudo-count (*p*) as the fifth percentile of the combined RPKM values of LmnB1 and Dam, then computed the scaled log2 seq ratio as log2 ((LmnB1 RPKM + *p*)/(Dam RPKM + *p*)). The ChIP-seq peaks were assigned to the same set of 2 kb windows based on the peak center and then used to depict their distribution relative to sLADs (Figure 6). The 2 kb window containing the TSS and TES of each mouse gene was determined in order to fetch the data upstream and downstream for gene-related analyses (Figure 4A, Figure 7, Additional file 2: Figure S3 and S4). All the above analyses were implemented by R and Perl scripts.

For the Spearman correlation test between Dam-chip and Dam-seq, the log2 chip ratio of each 60 bp probe was assigned to the above set of 2 kb windows by the probe location. One probe may overlap with and thus be assigned to two adjacent windows, and values of multiple probes in the same window were averaged. Then the Spearman correlation coefficient was calculated between the log2 chip ratios and the log2 seq ratios.

For the analyses of sequencing depths and sLADs concordance (Figure 1B-C), samples of various sizes (each with three replicates) were generated from the entire dataset of MEF LmnB1 by the “sample” function in R. The same set of SICER parameters as described earlier was used for sLAD identification.

### Preparation of the mouse gene list and expression data

First, we downloaded tables named knownGene, kgXref, knownToLocusLink from UCSC Table Browser (http://genome.ucsc.edu/cgi-bin/hgTables?command=start; [41]) on May 21, 2012 and merged the three tables by the known gene IDs, which resulted in a list of 55105 transcripts. Second, after grouping transcripts with the same gene symbol, we removed incompatible cases in which transcripts are located in both plus and minus strands or in which some shorter transcripts have no overlaps with the longest transcript. Finally, we retained the longest transcripts for each gene symbol for further analyses. The resulted gene list contains 29235 genes on chromosome 1–19 and X.

For a subset of 29235 genes (19269 genes), expression data of myoblasts are available from GEO database under the accession number GSE19968 [32]. We downloaded supplementary files for the three replicates of C2C12 myoblast expression profiling arrays (GSM499013-15) which contain the processed array data by Agilent Feature Extraction Version 10.1.1 [32]. Based on the online instruction (http://www.genomics.agilent.com/files/Manual/G3300-90001_FE_Plugin_A1.pdf), the data from an array probe contained a number of GeneSpring flags, each of which had three levels: Absent (A), Marginal (M) or Present (P). We identified a transcript (detected by a probe) as present only when all flags were shown as P. A gene was considered as expressed only when the transcripts were present in all three replicates of arrays. Custom Perl scripts were written to implement the task. The parsed expression states were used in Figure 4A, Figure 7, and Additional file 2: Figure S3 and Figure S4.

### Immunostaining and DNA immuno-FISH

Immunostaining of histone modifications, DNA immuno-FISH and imaging analyses were carried out in C2C12 myoblasts as described previously [23]. Antibodies used are the following: anti-LmnB (Santa Cruz, sc-6217), anti-H3K4me1 (Abcam, ab8895), anti-H3K4me2 (Abcam, ab32356), anti-H3K4me3 (Abcam, ab8580), anti-H3K9Ac (Abcam, ab4441), anti-H3K9me2 (Millipore, 07–441), anti-H3K27me3 (Millipore, 07–449), anti-H3K36me3 (Abcam, ab9050) and anti-H3K79me2 (Abcam, ab3594).

## Abbreviations

NL: Nuclear lamina; DamID: DNA adenine methyltransferase identification; sLAD: Sequencing-based lamina-associated domain; INM: Inner nuclear membrane; LAD: Lamina-associated domain; Pol II: RNA polymerase II; TSS: Transcription start site; NGS: Next generation sequencing; MEF: Mouse embryonic fibroblast; Dam: DNA adenine methyltransferase; FISH: Fluorescence in situ hybridization; TES: Transcription end site; FDR: False discovery rate; RPKM: Reads per kilobase per million mapped reads

## Competing interests

The authors declare that they have no competing interests.

## Authors’ contributions

FW and JY designed experiments. FW performed DamID assay, analyzed sequencing data and performed all the bioinformatic analyses. JY performed cell culture, lentivirus infection, FISH, immunostaining and imaging analysis. FW and JY wrote the paper. All authors read and approved the final manuscript.

## Supplementary Material

Additional file 1: Table S1 Genomic coordinates of identified sLAD regions and non-sLAD regions in MEFs. **Table S2**. Genomic coordinates of identified sLAD regions and non-sLAD regions in myoblasts. **Table S3**. The list of sLAD genes and non-sLAD genes in MEFs and myoblasts. **Table S4**. Comparison of myoblast and MEF gene expression states within common sLADs and cell type-specific sLADs. Click here for file

Additional file 2: Figure S1 The DamID maps of mouse chromosome 1–19 and X in MEFs and myoblasts. **Figure S2**. Genome browser tracks displaying representative genomic regions that are identified as sLADs but not LADs (A-C) or LADs but not sLADs (D, E) in 3T3 fibroblasts. **Figure S3**. Dam and LmnB1 read densities across genic regions in myoblasts. **Figure S4**. Distributions of histone modifications and Pol II across genic regions in myoblasts. **Figure S5**. A 6 Mb segment in chromosome 1 shows that the differential sLAD regions between MEFs and myoblasts include genes. **Figure S6**. Gene enrichment analysis of sLAD genes. Click here for file

Additional file 3 Comparisons of sLADs and sLAD Genes between MEFs and Myoblasts. Click here for file
