# Supplementary material for: Spatial compartmentalization at the nuclear periphery characterized by genome-wide mapping
Source: BMC Genomics. 2013 Aug 30;14:591. doi: 10.1186/1471-2164-14-591 (PMC3849850; doi:10.1186/1471-2164-14-591)

Supplemental Figure S1. The DamID maps of mouse chromosome 1-19 and X in MEFs and myoblasts. Figure legends follow Figure 1.

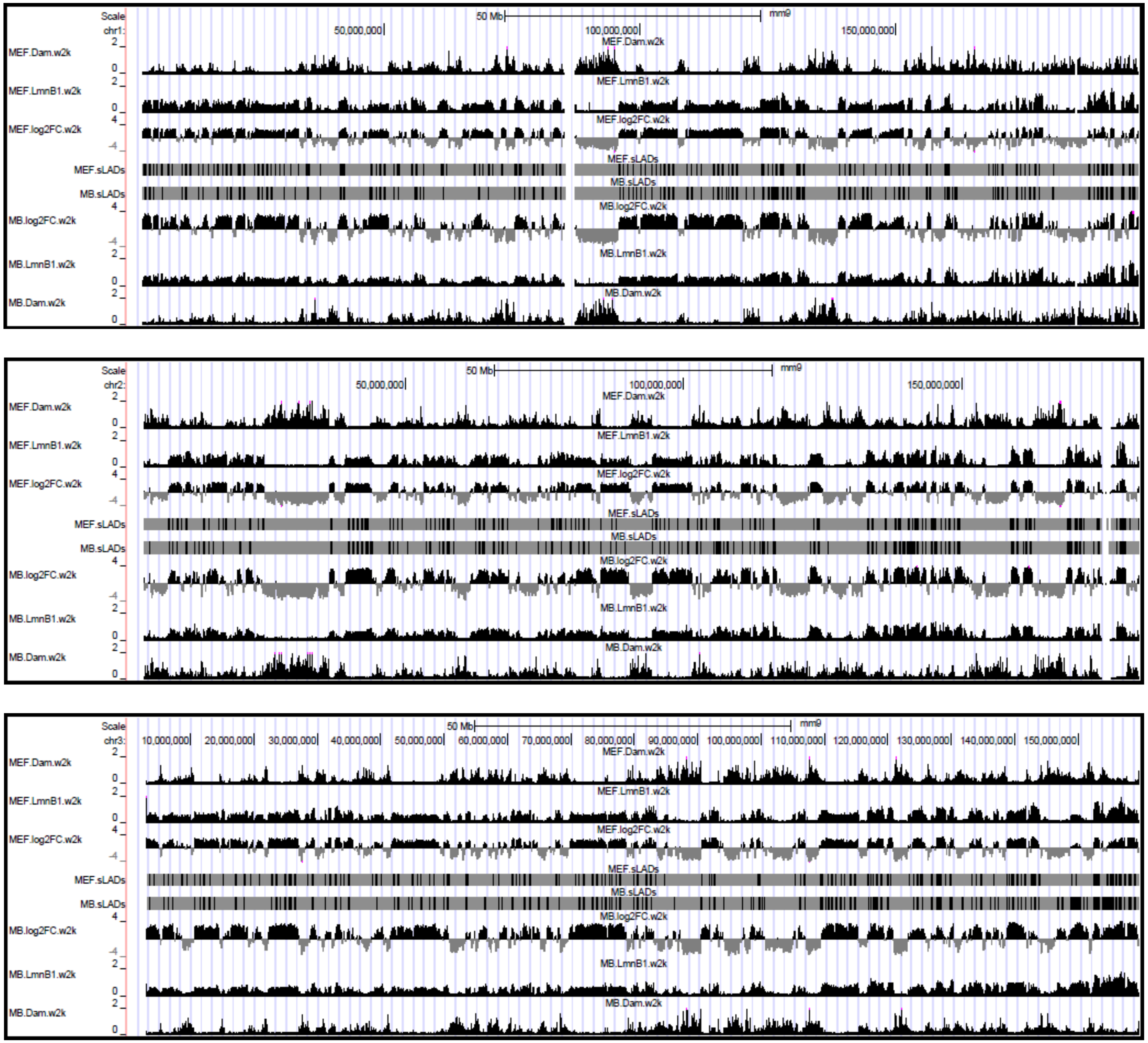

Suppl. Fig. S1 continued (Wu et al)

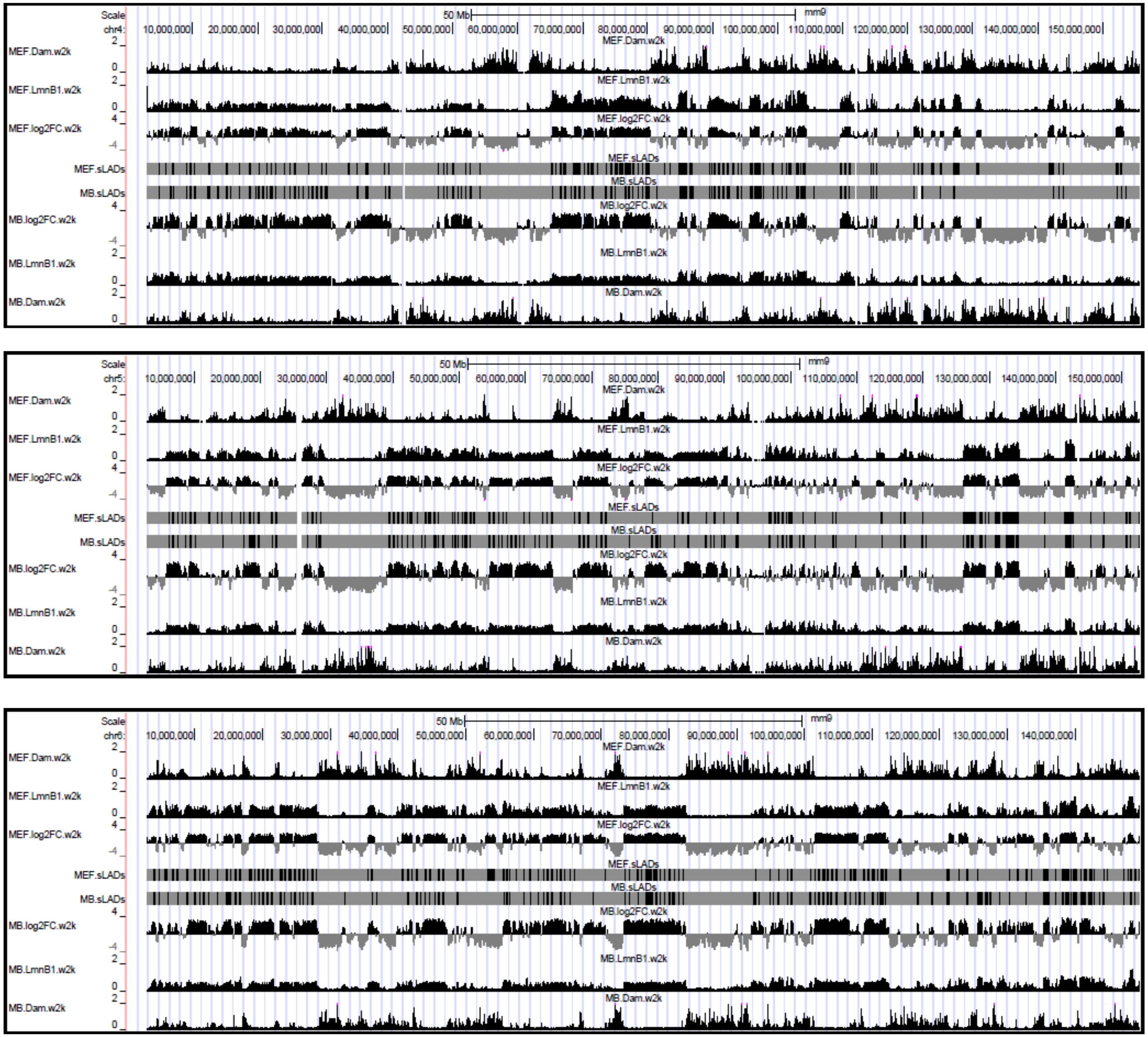

Suppl. Fig. S1 continued (Wu et al)

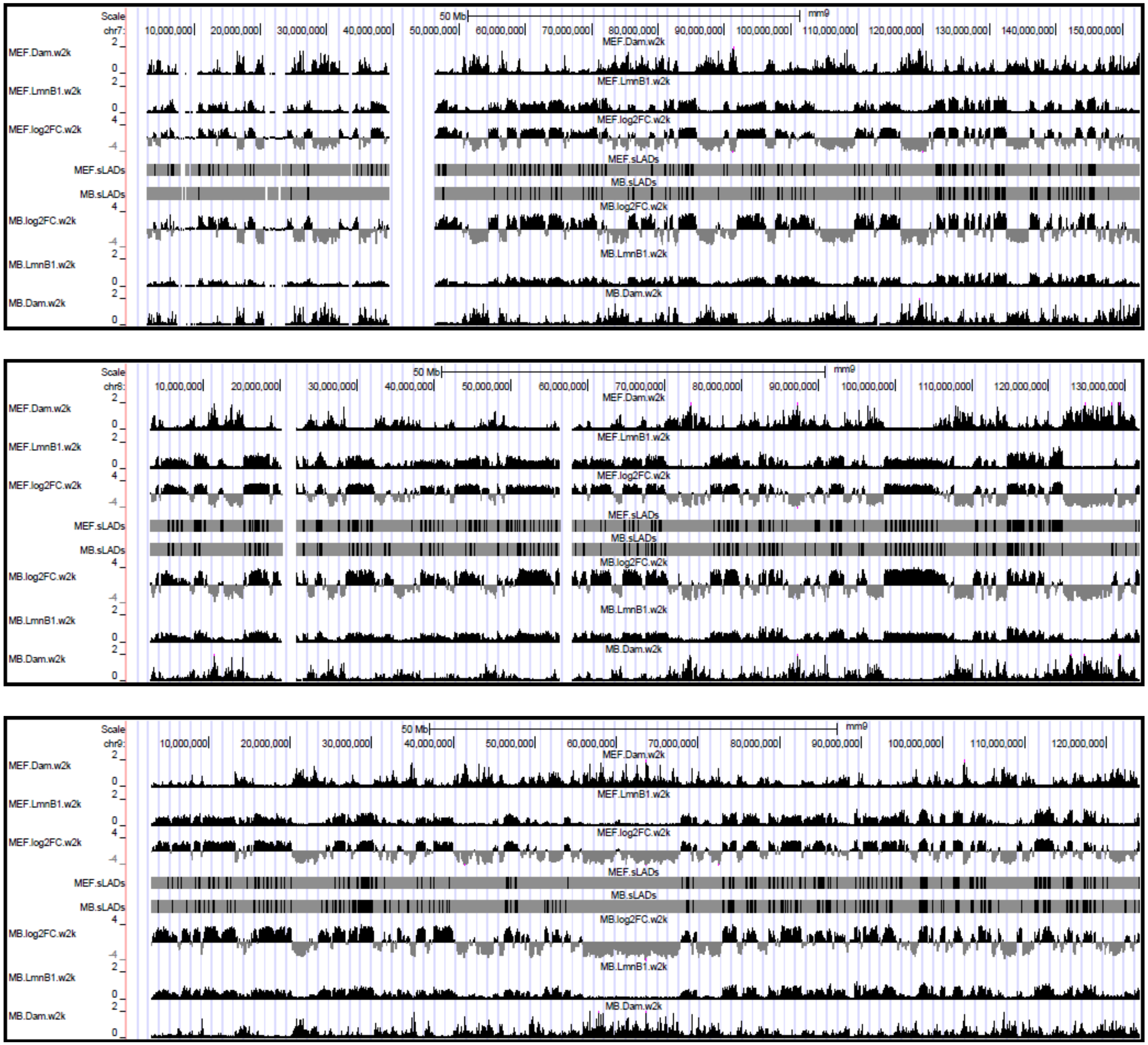

Suppl. Fig. S1 continued (Wu et al)

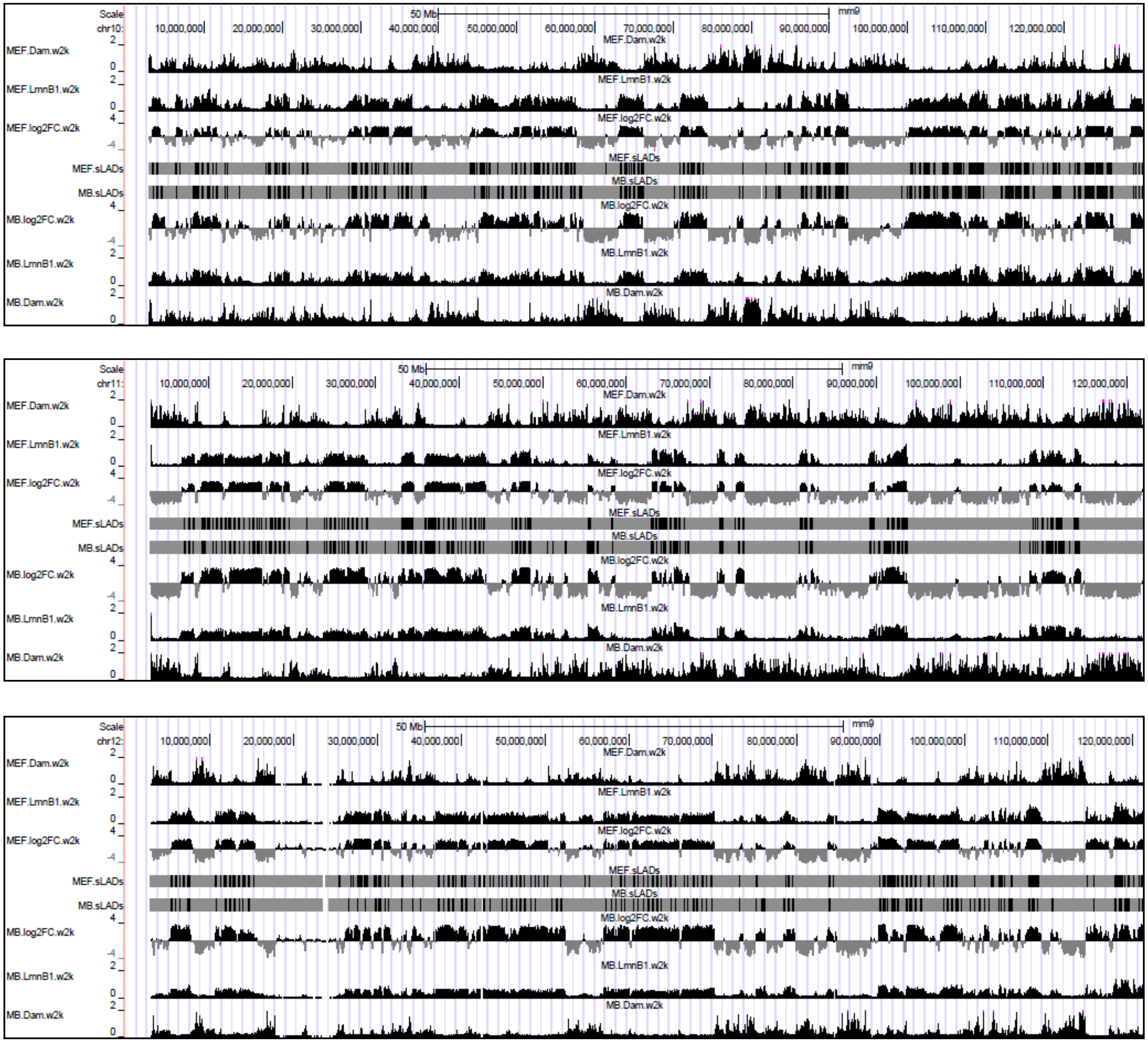

Suppl. Fig. S1 continued (Wu et al)

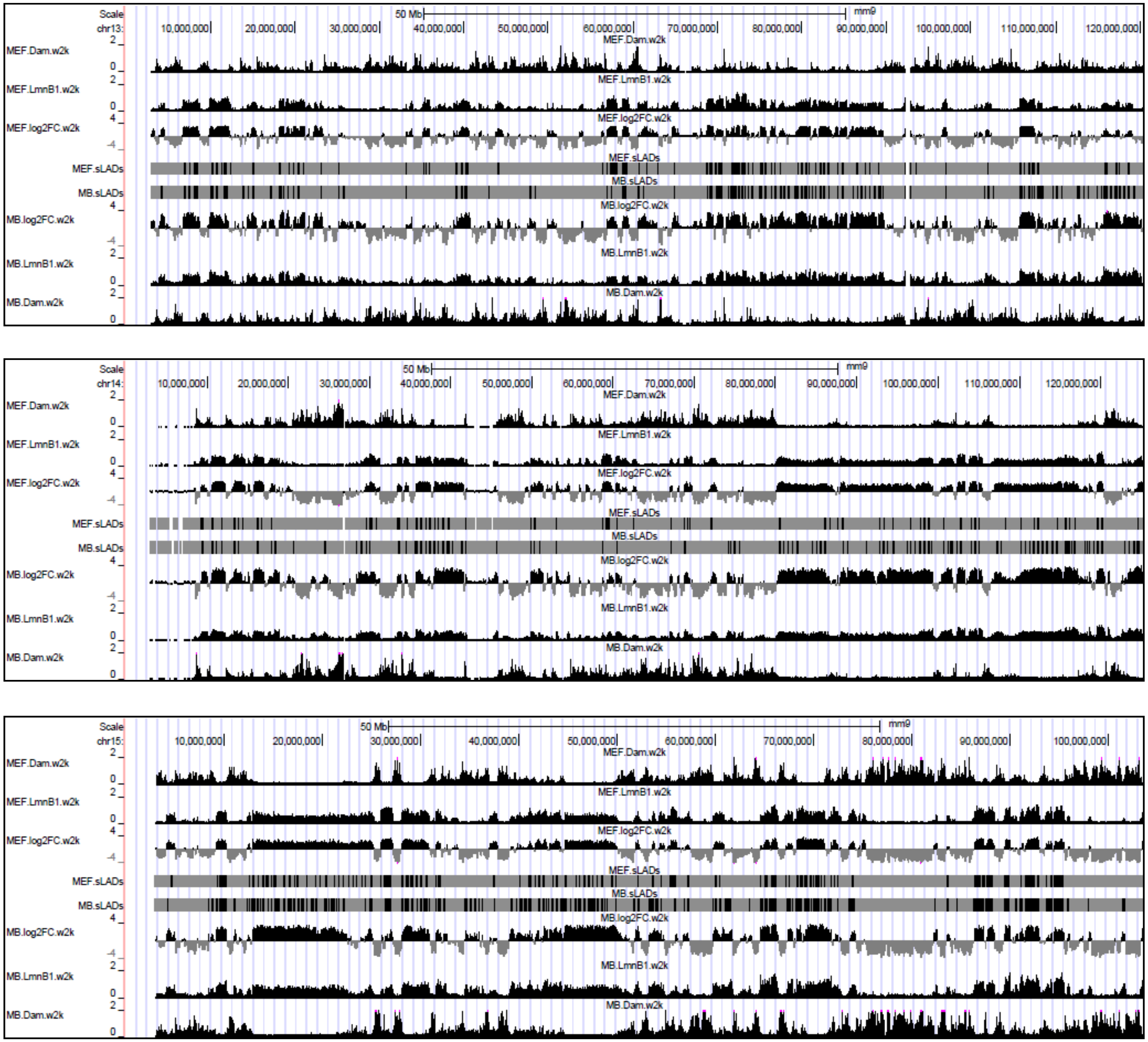

Suppl. Fig. S1 continued (Wu et al)

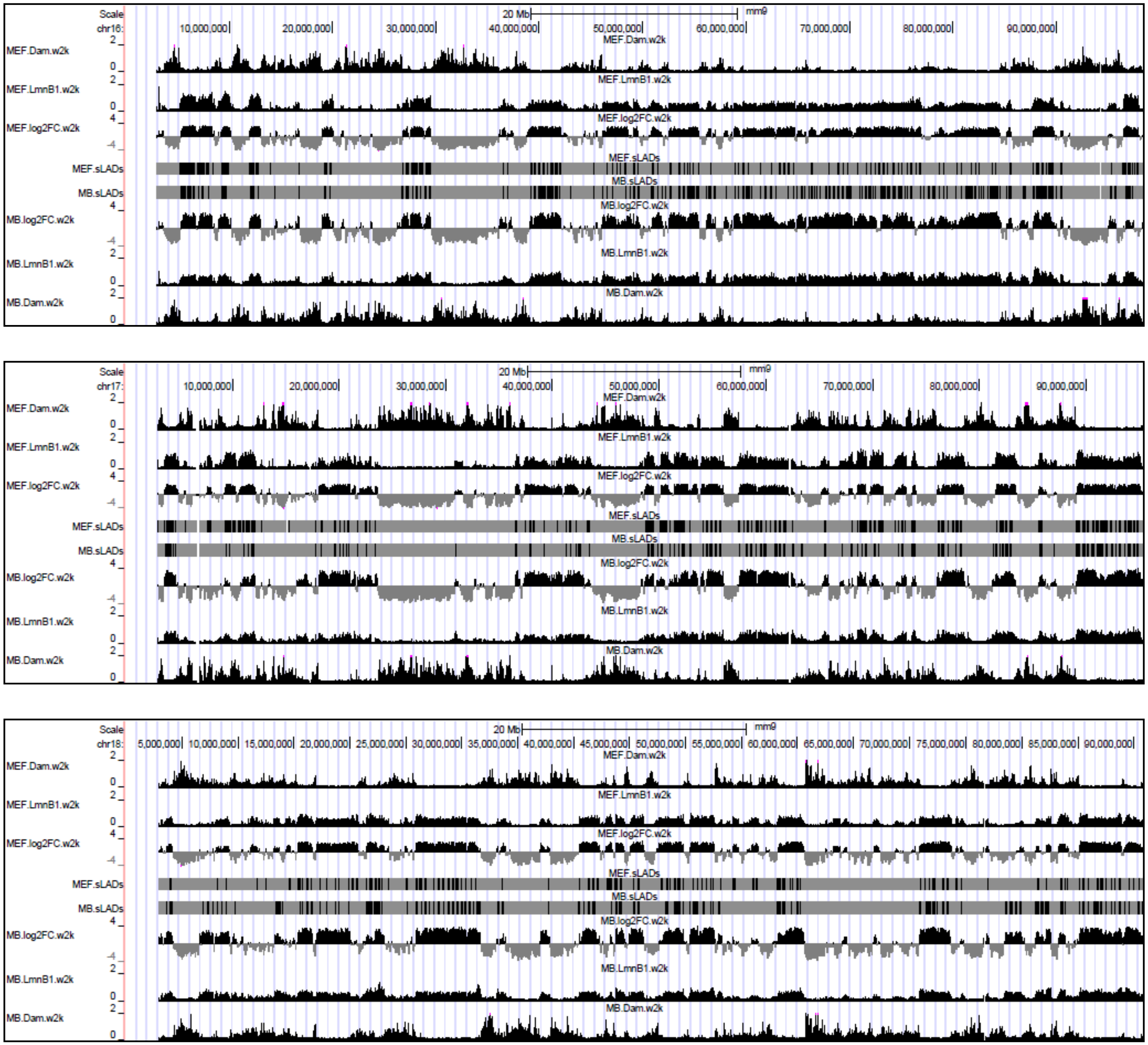

Suppl. Fig. S1 continued (Wu et al)

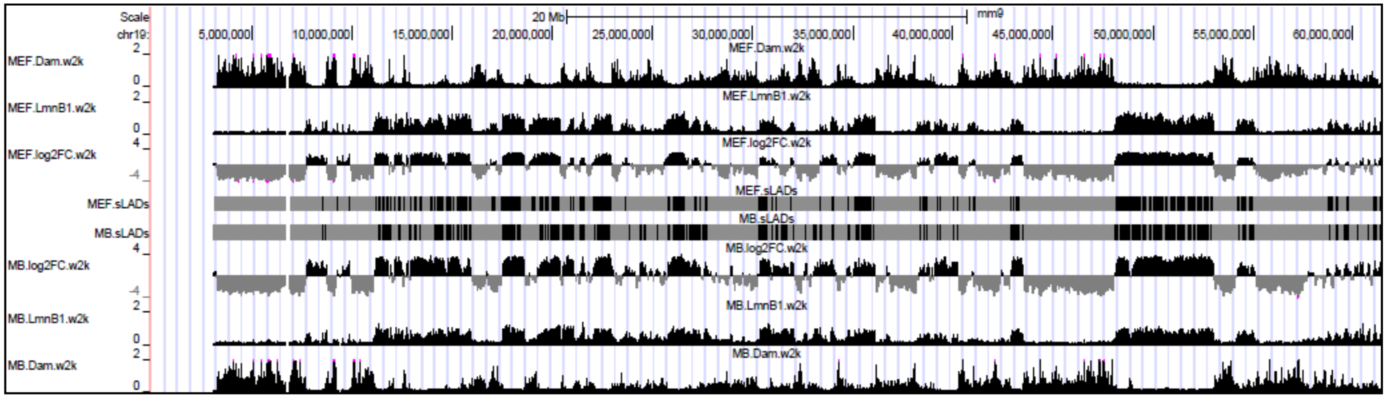

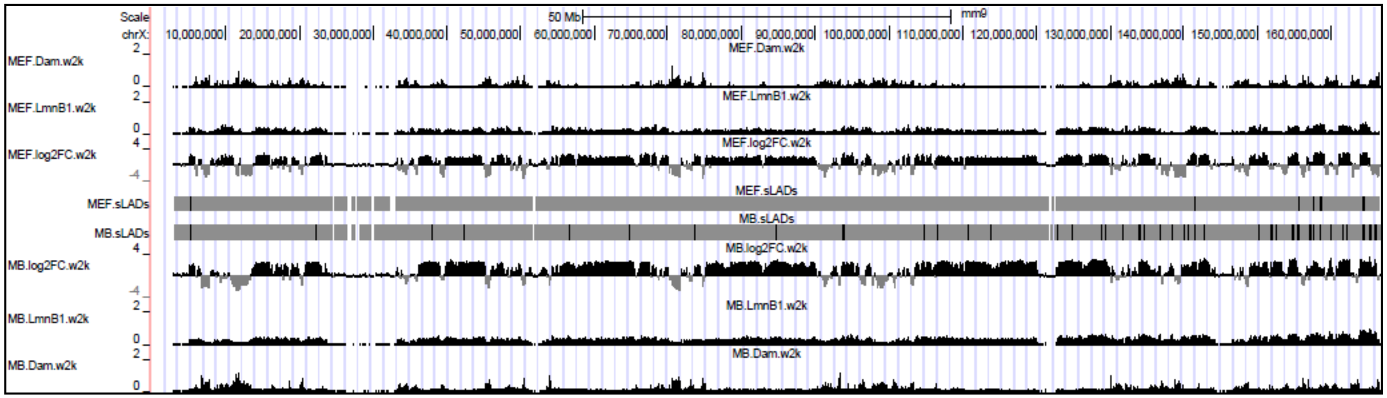

Supplemental Figure S2. Genome browser tracks displaying genomic regions that are identified as sLADs but not LADs (A-C) or LADs but not sLADs (D, E) in 3T3 fibroblasts. Tracks are described as in Figure 1. The positions of BACs used as DNA-FISH probes (images shown in Figure 3C) are noted as red bars.

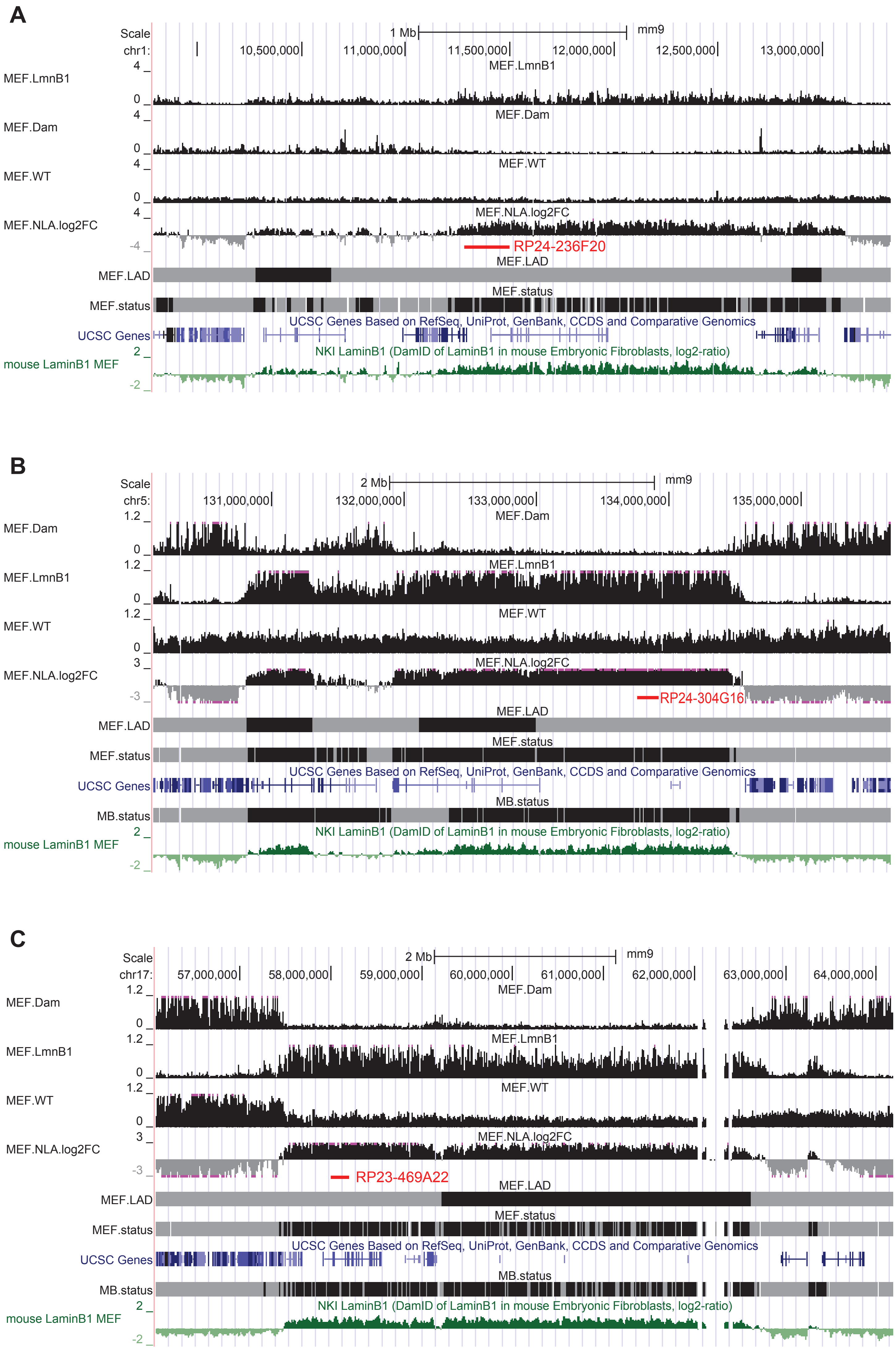

Suppl. Fig. S2, continued (Wu et al)

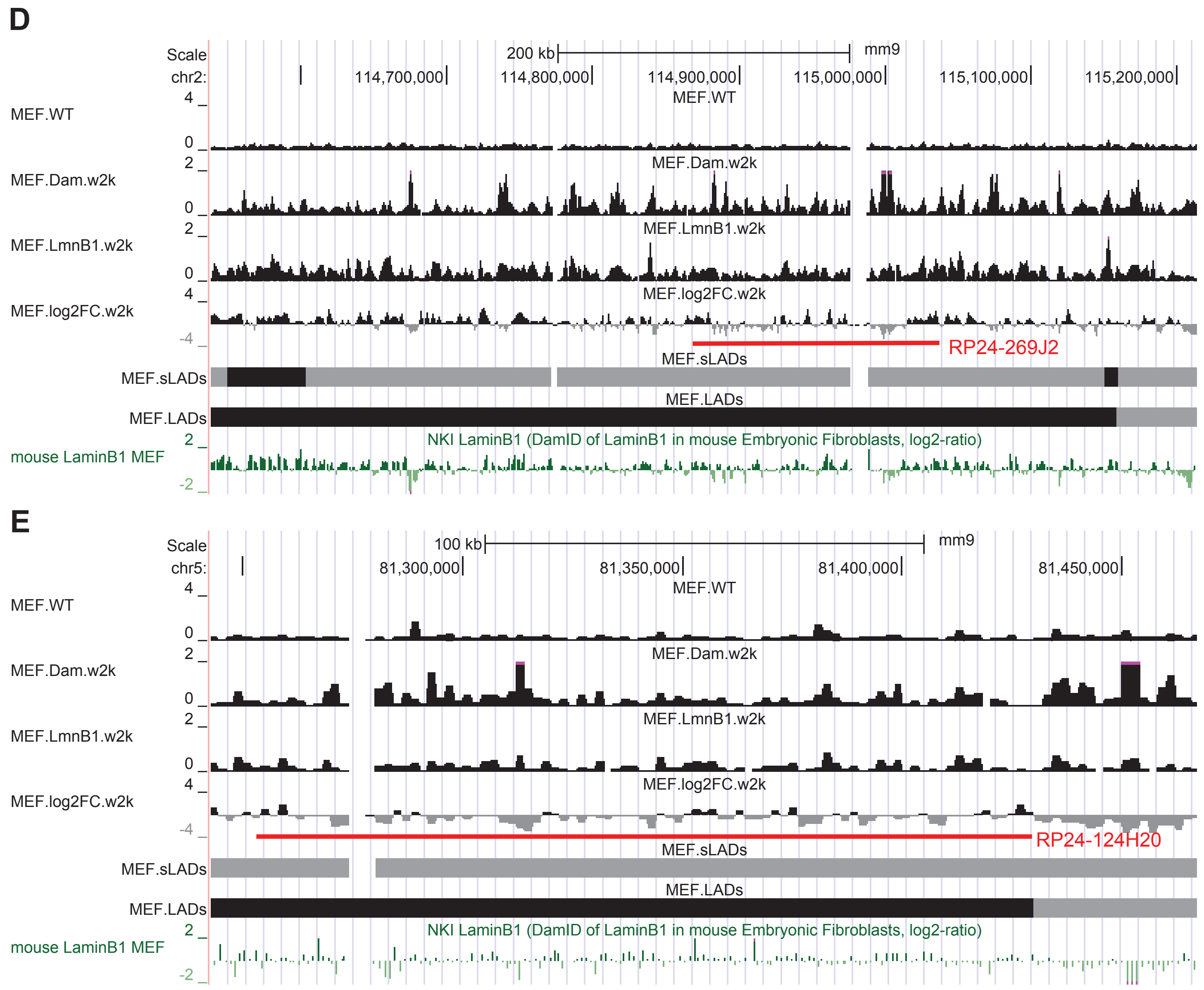

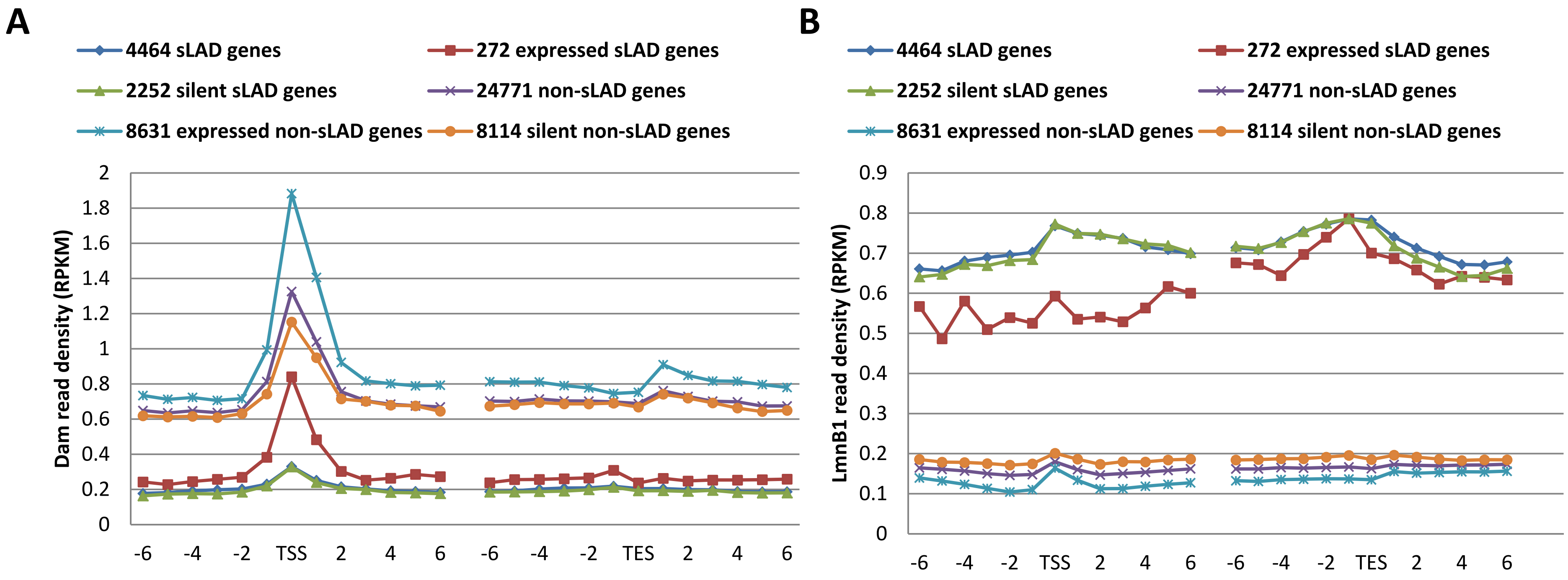

Supplemental Figure S3. Dam and LmnB1 read densities across genic regions in myoblasts. Read densities (reads per kilo base per million mapped reads, RPKM) across different genes were aligned using the windows containing TSS and TES respectively, and the average densities from six windows upstream to six windows downstream were plotted.

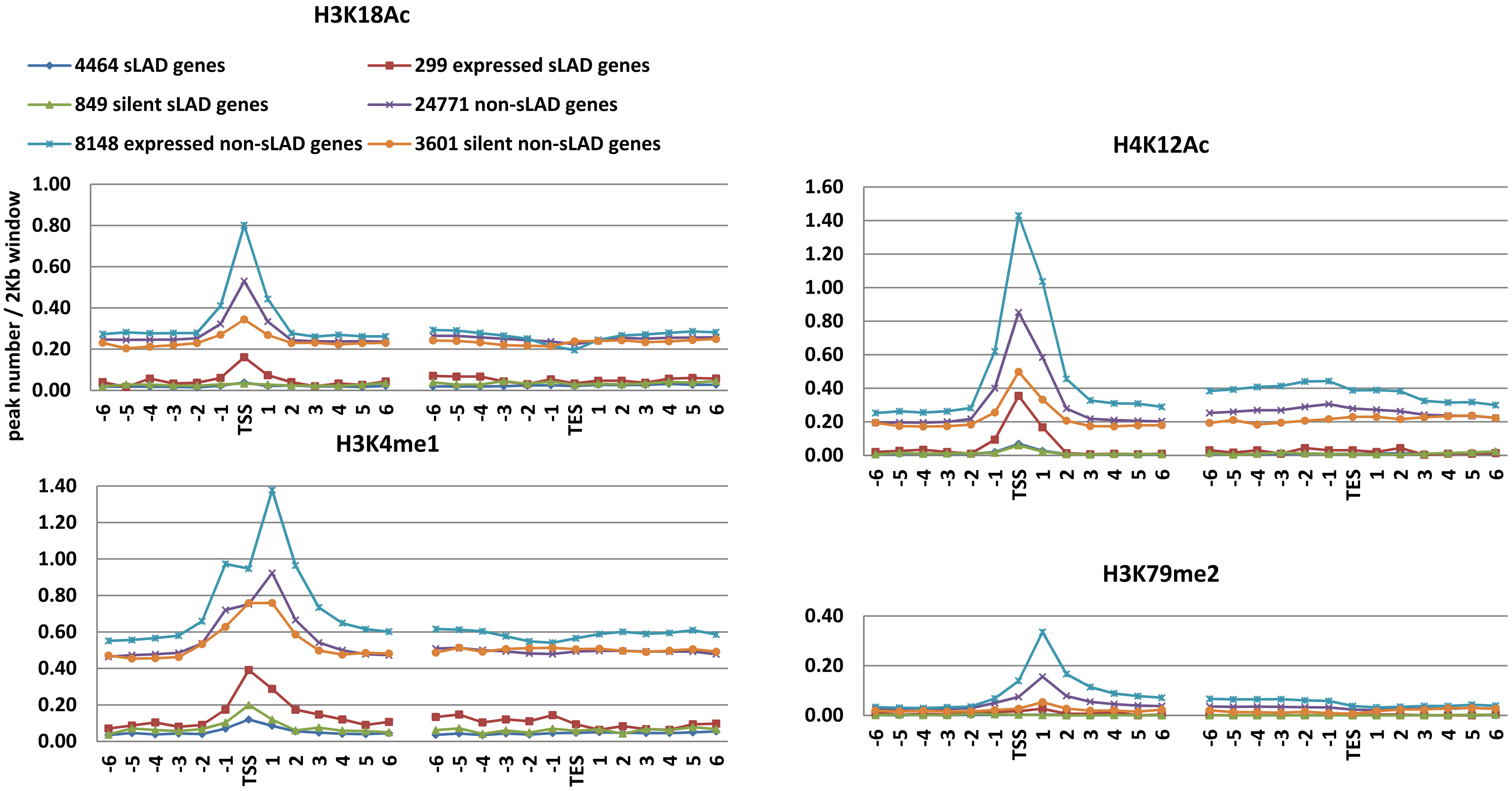

Supplemental Figure S4. Distributions of histone modifications and Pol II across genic regions in myoblasts.
Figure legends follow Figure 7.

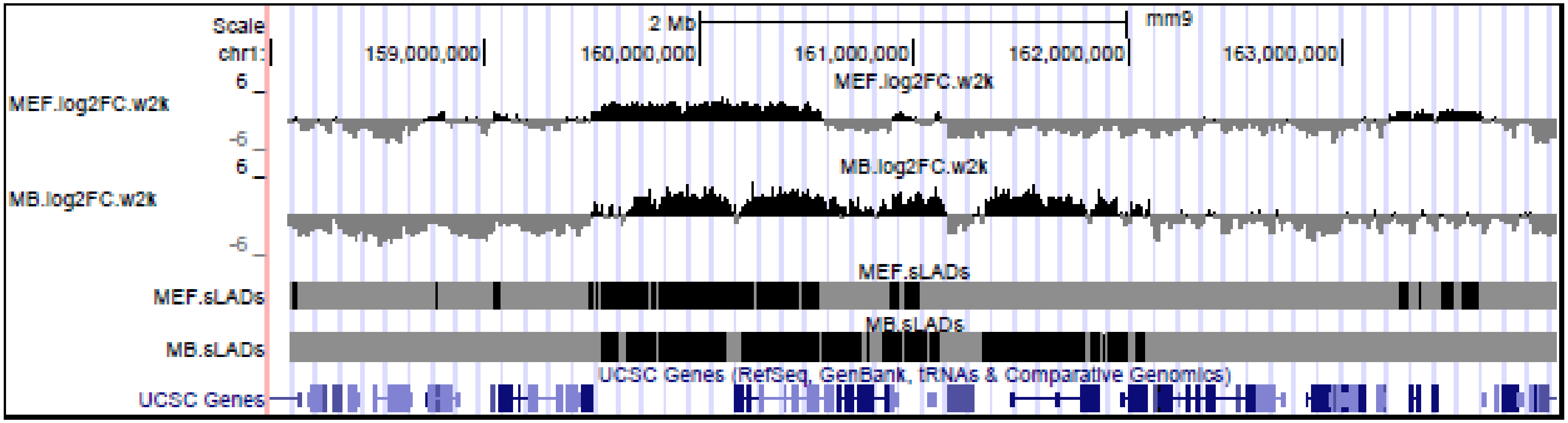

Supplemental Figure S5. A 6Mb segment in chromosome 1 shows that the differential sLAD regions between MEFs and myoblasts include genes.

Supplemental Figure S6. Gene enrichment analysis of sLAD genes. (A) myoblast specific sLAD genes, **(B)** MEF specific sLAD genes and **(C)** common sLAD genes between MEFs and myoblasts. Only GO categories with FDR less than 0.01 are shown.

A

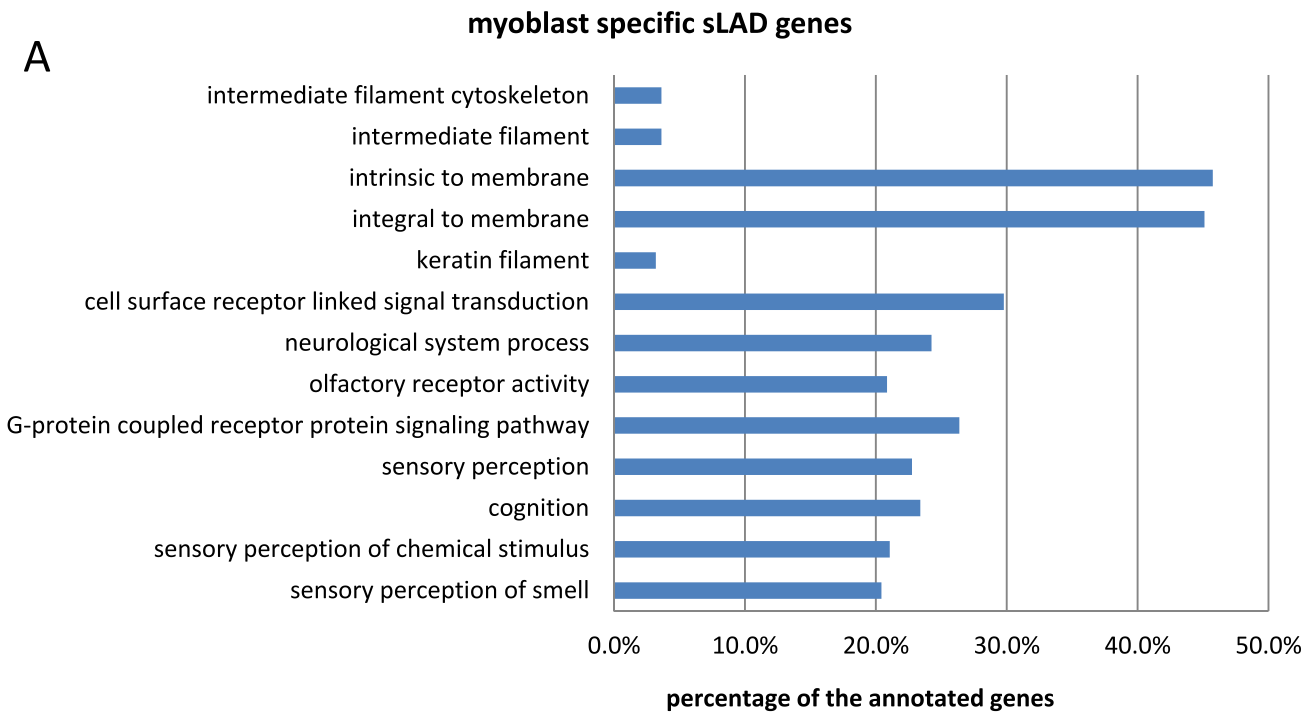

Suppl. Fig. S6 continued (Wu et al)

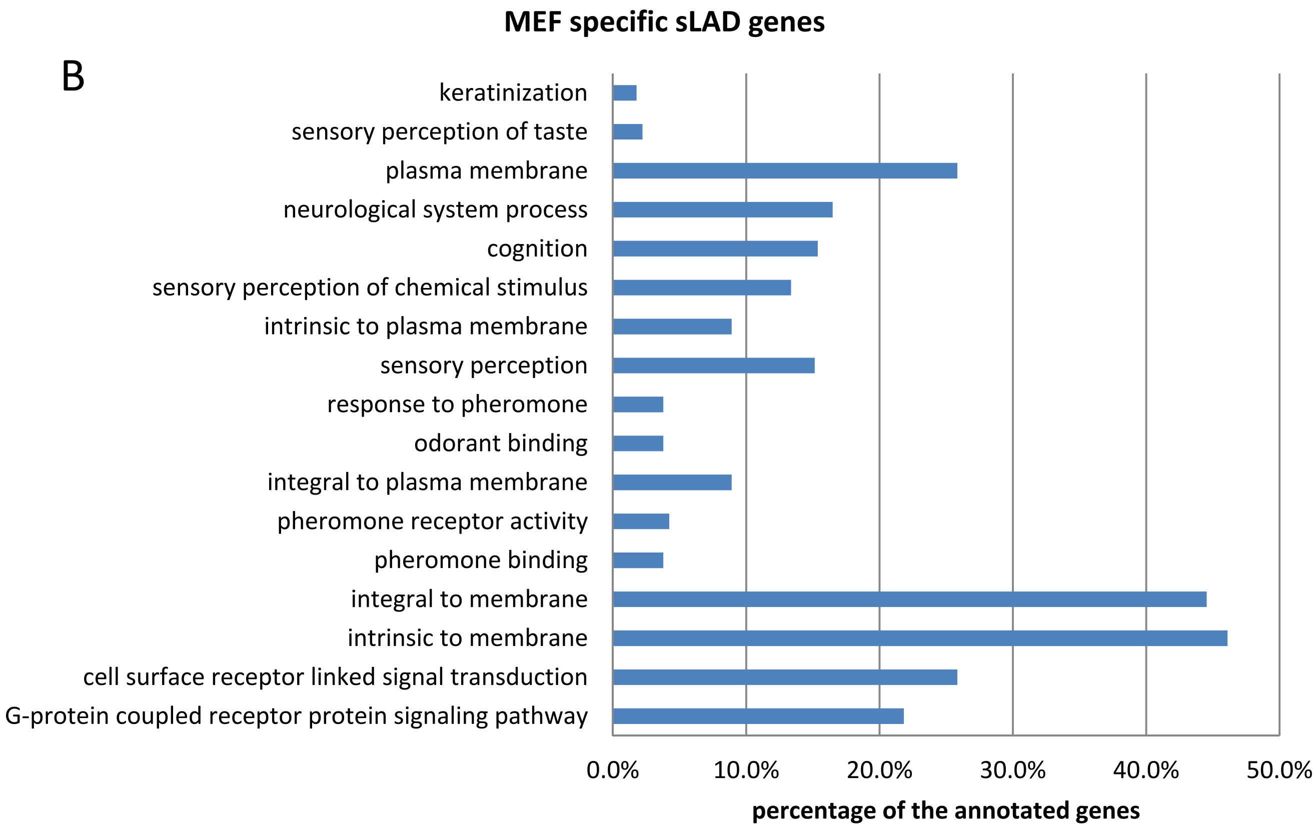

Suppl. Fig. S6 continued (Wu et al)

C

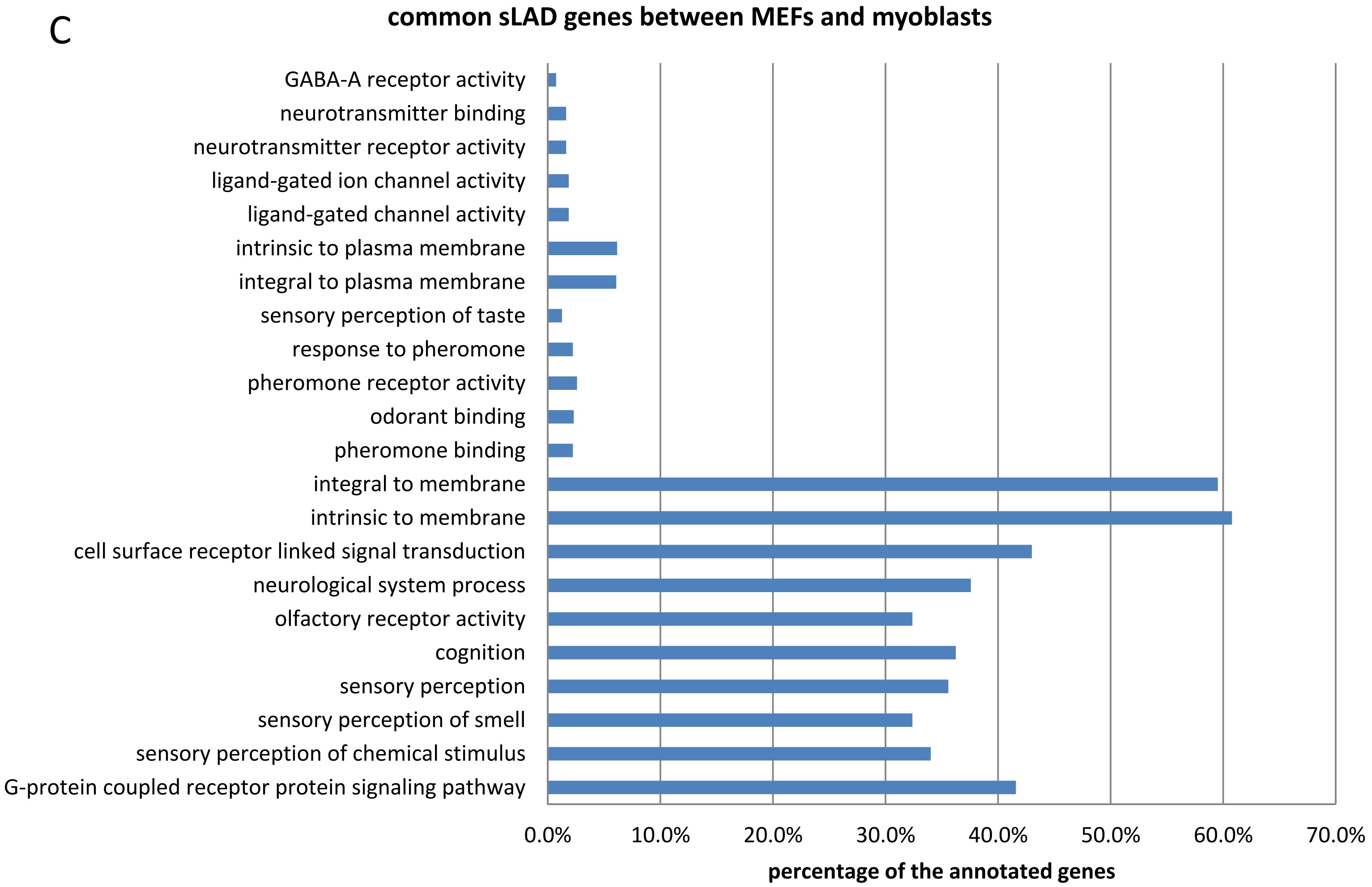

Supplement: Additional file 2: Figure S1 — The DamID maps of mouse chromosome 1–19 and X in MEFs and myoblasts. Figure S2. Genome browser tracks displaying representative genomic regions that are identified as sLADs but not LADs (A-C) or LADs but not sLADs (D, E) in 3T3 fibroblasts. Figure S3. Dam and LmnB1 read densities across genic regions in myoblasts. Figure S4. Distributions of histone modifications and Pol II across genic regions in myoblasts. Figure S5. A 6 Mb segment in chromosome 1 shows that the differential sLAD regions between MEFs and myoblasts include genes. Figure S6. Gene enrichment analysis of sLAD genes. [file 1471-2164-14-591-S2.pdf]
