# Supplementary material for: Spatial compartmentalization at the nuclear periphery characterized by genome-wide mapping
Source: BMC Genomics. 2013 Aug 30;14:591. doi: 10.1186/1471-2164-14-591 (PMC3849850; doi:10.1186/1471-2164-14-591)
Supplement: Additional file 3 — Comparisons of sLADs and sLAD Genes between MEFs and Myoblasts. [file 1471-2164-14-591-S3.pdf]

Spatial Compartmentalization at the nuclear periphery characterized by genome-wide mapping

Feinan Wu and Jie Yao

Additional file 3.

Supplemental Results

Comparisons of sLADs and sLAD Genes between MEFs and Myoblasts

Between MEF and myoblast cell lines, sLADs distributions are generally similar in most chromosomes (Figure 1A and Additional file 1: Figure S1) but differ when viewed at a higher resolution (Additional file 1: Figure S5). As listed in Table 1, there are 14828 sLADs in myoblasts and 14737 in MEFs, of which 3179 (122.1 Mb, corresponding to 14.2% of the myoblast sLADs) are specific to myoblasts and 2802 (94.3 Mb, corresponding to 11.4% of the MEF sLADs) specific to MEFs. To examine whether these differential distributions of sLADs are linked to differential gene expression, we used Fisher's exact test to compare the gene expression states between the two cell types for each of the following three gene groups: genes overlapping with common sLADs between myoblasts and MEFs (group 1), genes overlapping with sLADs specific to myoblasts (group 2) and genes overlapping with sLADs specific to MEFs (group 3). As shown in Additional file 2: Table S4, gene expression states in group 1 do not significantly differ between myoblasts and MEFs (two-sided p-value 0.19), while MEFs have a higher proportion of expressed genes in group 2 (one-sided p-value 0.001) but a lower proportion

of expressed genes in group 3 (one-sided p-value <0.0001) compared with myoblasts. These results suggest that cell type specific sLADs are correlated with the differential gene expression between myoblasts and MEFs.

Regarding sLAD genes, the two cell types share 3046 sLAD genes while 1418 and 1014 are specific to myoblasts and MEFs respectively. Next we have performed some preliminary gene enrichment analyses using DAVID [1, 2] on these three gene sets. As shown in Additional file 1: Figure S6, genes in some GO categories are enriched in all three datasets, such as membrane related, sensory perception, cognition and neurological system process. Since genes in these categories are not likely to function in fibroblasts and myoblasts, their enrichment in sLAD regions are consistent with the repressive roles of sLADs. Because only about half of the sLAD genes have annotations available in DAVID database, the gene enrichment analysis is not complete at this time. A more comprehensive, revealing analysis in the future will require combination of a complete gene annotation database, genome-wide gene expression data and sLADs information in a related cell type, such as terminally differentiated myotubes.

Supplemental Methods

Preparation of gene expression data of MEFs

In total 17189 out of 29235 genes have expression data in 3T3 MEFs available from GEO database under accession number GSE4739 [3]. We downloaded the data tables of the three replicates (GSM107391-93) of MEF expression profiling arrays, from which genes were considered as expressed only if they were detected as present ("P" in the field "ABS_CALL") in all three replicates. As a result, there were 8680 expressed genes and 8509 silent genes.

Gene enrichment analysis of sLAD genes

The selected subsets of sLAD genes as described in "Supplemental Results" were analyzed using the online DAVID tools (http://david.abcc.ncifcrf.gov/tools.jsp) with the 29235 genes as background. We chose DAVID-created Gene Ontology term set (including "GOTERM_BP_FAT", "GOTERM_CC_FAT" and "GOTERM_MF_FAT") and the "highest" stringency for functional annotation clustering, and presented only the categories with FDR < 0.01 (Additional file 1: Figure S6).

Supplemental References

1. Huang da W, Sherman BT, Lempicki RA: Bioinformatics enrichment tools: paths toward the comprehensive functional analysis of large gene lists. *Nucleic Acids Res* 2009, 37(1):1-13.

2. Huang da W, Sherman BT, Lempicki RA: Systematic and integrative analysis of large gene lists using DAVID bioinformatics resources. *Nat Protoc* 2009, 4(1):44-57.

3. Yamamoto T, Ebisuya M, Ashida F, Okamoto K, Yonehara S, Nishida E: Continuous ERK activation downregulates antiproliferative genes throughout G1 phase to allow cell-cycle progression. *Curr Biol* 2006, 16(12):1171-1182.
